# Associations between the timing of 24 h physical activity and diabetes mellitus: results from a nationally representative sample of the US population

**DOI:** 10.1007/s00125-025-06368-9

**Published:** 2025-02-21

**Authors:** Qian Xiao, Qiuyu Feng, Martin K. Rutter, Gali Albalak, Heming Wang, Raymond Noordam

**Affiliations:** 1 Department of Epidemiology, School of Public Health, The University of Texas Health Science Center at Houston, Houston, TX USA; 2 Center for Spatial‑temporal Modeling for Applications in Population Sciences, School of Public Health, The University of Texas Health Science Center at Houston, Houston, TX USA; 3 Department of Human Genetics, Leiden University Medical Center, Leiden, the Netherlands; 4 Centre for Biological Timing, Faculty of Biology, Medicine and Health, University of Manchester, Manchester, UK; 5 Diabetes, Endocrinology and Metabolism Centre, Manchester University NHS Foundation Trust, Manchester Academic Health Science Centre, Manchester, UK; 6 NIHR Manchester Biomedical Research Centre, Manchester, UK; 7 Department of Internal Medicine, Section of Gerontology and Geriatrics, Leiden University Medical Center, Leiden, the Netherlands; 8 Division of Sleep and Circadian Disorders, Brigham and Women’s Hospital, Harvard Medical School, Boston, MA USA; 9 Health Campus the Hague/Public Health and Primary Care, Leiden University Medical Center, the Hague, the Netherlands

**Keywords:** Chronotype, Diabetes, Physical activity, Timing, Type 2 diabetes

## Abstract

**Aims/hypothesis:**

Growing evidence suggests that timing may be an important aspect of physical activity that influences cardiometabolic health. However, the current literature is inconclusive regarding the time of day that physical activity offers the greatest metabolic advantages. We investigated associations between hourly physical activity levels and diabetes mellitus and glycaemic biomarkers in a cross-sectional and nationally representative sample of US adults.

**Methods:**

We studied 7074 adults (mean age 48 years; 52% women) from the National Health and Nutrition Examination Survey (2011–2014). Physical activity was measured by actigraphy. A monitor-independent movement summary (MIMS) unit was used to derive the total activity level (divided into quintiles) for hourly windows that were defined relative to sleep timing and according to clock time. The primary outcome was prevalent diabetes, and secondary outcomes included fasting glucose, fasting insulin, HOMA-IR and 2 h OGTT results.

**Results:**

Physical activity levels in late morning and late afternoon were associated with lower adjusted odds of diabetes. Specifically, in late morning (8:01–9:00 h after the sleep midpoint), the highest quintile of activity was associated with a 35% decrease (OR 0.65; 95% CI 0.44, 0.96) in the odds of diabetes when compared with the lowest quintile, while in late afternoon (11:01–17:00 h after the sleep midpoint), the highest quintiles were associated with 56% and 36% lower odds (OR 0.44; 95% CI 0.29, 0.69 and OR 0.64; 95% CI 0.43, 0.95). Higher night-time activity was associated with higher odds of diabetes. Similar patterns of results were observed with OGTT data and across subgroups of age, gender, race/ethnicity, chronotype and sleep duration.

**Conclusions/interpretation:**

Our findings suggest that the timing of physical activity may modulate its metabolic effects.

**Graphical Abstract:**

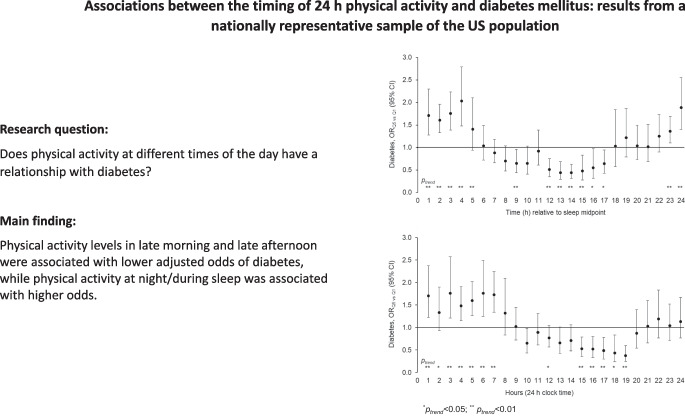

**Supplementary Information:**

The online version of this article (10.1007/s00125-025-06368-9) contains peer-reviewed but unedited supplementary material.


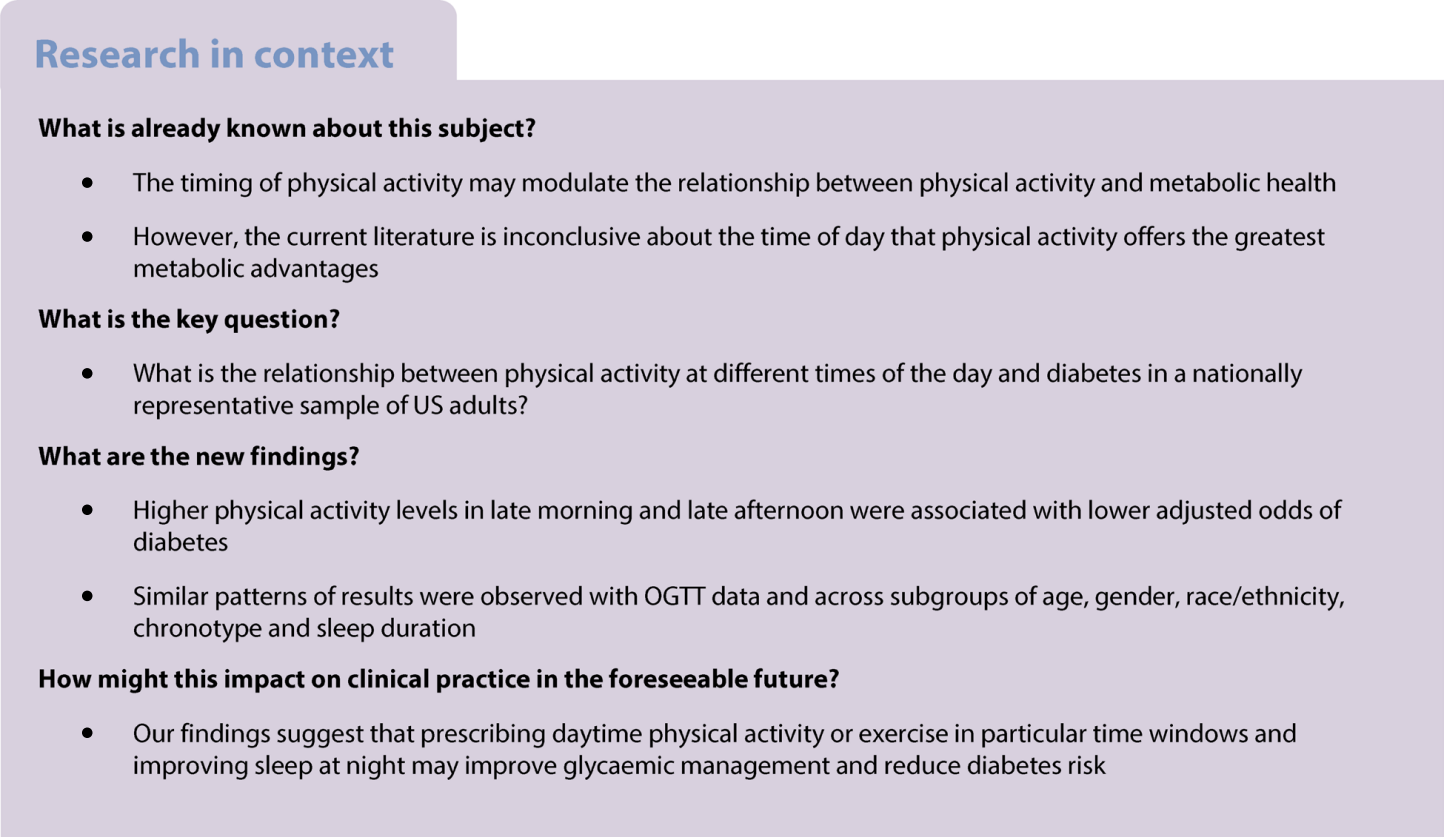


## Introduction

Diabetes mellitus is a serious and common chronic condition that has emerged as a major global public health threat. The International Diabetes Federation has estimated that 10.5% of the global population aged 20–79 years, or 536.6 million people, had diabetes in 2021 [1]. In the USA, the age-standardised prevalence of type 2 diabetes mellitus increased from 9.8% in 1999–2000 to 14.3% in 2017–2018 [2]. Decades of research on type 2 diabetes mellitus have specifically identified multiple modifiable lifestyle factors that play an important role in disease risk and management, including obesity, unhealthy dietary patterns, physical inactivity and suboptimal sleep [3, 4].

Physical activity has long been recognised as a critical component in type 2 diabetes mellitus prevention and treatment programmes, and its metabolic benefits are at least partially independent of its impact on body weight [5, 6]. Traditionally, research on physical activity and diabetes has focused on the volume, intensity, mode of exercise and duration. More recently, there has been growing interest in investigating whether timing may modulate the metabolic advantages of physical activity [7–15]. However, findings from these studies are mixed, and have generally involved small study samples. Some studies suggested that being physically active in the afternoon or later during the day may be associated with stronger metabolic improvement [7, 10, 12, 14], while another study suggested that both early morning and late afternoon physical activity were associated with favourable metabolic outcomes [15]. However, other studies showed no relationship between physical activity timing and diabetes [8, 11, 13]. Moreover, one study also suggested that the association between timing of physical activity and cardiovascular health in people with diabetes may differ by gender [9]. Recent reviews and meta-analyses concluded that there is insufficient evidence to recommend physical activity at a particular time of the day [16, 17].

In addition to the mixed findings, we have identified several gaps in the existing research that warrant further investigations. First, most study samples had insufficient demographic diversity to enable group-specific analysis and enhance generalisability. Second, previous studies defined timing using broad windows that did not provide sufficient temporal granularity and may have contributed to the inconsistent findings. Third, most of the studies did not consider the potential role of sleep timing and/or chronotype, which may both be confounders due to their link with both physical activity patterns and diabetes risk [18, 19], and an effect modifier as suggested by several epidemiological studies focusing on chrononutrition [20, 21].

To address these gaps, we studied cross-sectional relationships between actigraphy-measured physical activity in hourly windows (based on both clock time and relative to sleep timing) and prevalent diabetes and multiple glycaemic markers (i.e. fasting glucose, fasting insulin, HOMA-IR and 2 h OGTT results) in a large, diverse and nationally representative sample in the USA.

## Methods

### Study population

**Fig. 1:**
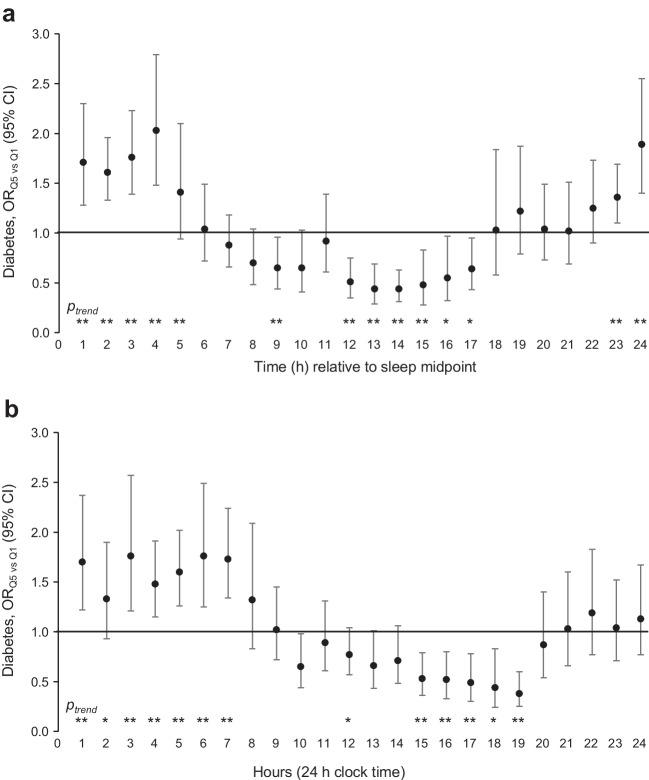
Associations between average hourly physical activity and prevalent diabetes among NHANES (2011–2014) participants, comparing the highest quintile of hourly activity with the lowest quintile. Each hourly window was determined relative to the average sleep midpoint of the individual (**a**) or by clock time (**b**). Associations are expressed as OR and 95% CI, comparing individuals in the highest activity quintile for each hourly window (Q5) with those in the lowest quintile (Q1), derived from multiple logistic regression models adjusted for age (continuous) and gender (men/women), race/ethnicity (non-Hispanic white, non-Hispanic black, Hispanic, others), education (less than high school, high school graduate, some college, college graduate or above), household income (<US$20k, US$20k–44.9k, US$45k–74.9k, ≥US$75k), marital status (married/not married), smoking (current smoker, former smoker, never smoker or fewer than 100 cigarettes in life), alcohol consumption (<1 drink/week, 1 drink/week – <1 drink/day, ≥1 drink/day), BMI (kg/m^2^: <18.5, 18.5 – <25, 25 – <30, ≥30), total energy intake (continuous), sleep duration (<7, 7–9, >9 h), sleep midpoint (continuous) and daily total physical activity (continuous). Diabetes was defined as HbA_1c_ ≥48 mmol/mol (6.5%) or self-reported diagnosis of diabetes. For the average sleep midpoint of the individual, each window is labelled by the number of hours passed beyond the sleep midpoint at the end of the hour. For example, the first hourly window started after the sleep midpoint and ended 60 min after that. For clock time, each window is labelled by the ending hour. For example, the first window was 00:01–01:00 hours. *p*_trend_ represents the *p* values for trend, with hourly physical activity modelled as a continuous variable (^*^p<0.05; ^**^p<0.01)

We used data from National Health and Nutrition Examination Survey (NHANES), a national survey that is designed to assess the health and nutritional status in a representative sample of non-institutionalised civilians (those who are not in the armed forces and who live in the community rather than a care home or hospital) in the USA. NHANES uses a four-stage probability sampling design, and oversamples racial/ethnic minority and low-income groups and adults aged 80 years and over. NHANES is conducted by the US National Center for Health Statistics and is approved by its ethics review board. The sample selection process is shown in electronic supplementary material (ESM) Fig. 1. A total of 19,931 participants were included in the NHANES 2011–2014 cycles, and actigraphy data were available for 14,511 of them. Of these, we excluded people who were aged $\le$ 20 years (*n*=5119), were pregnant (*n*=102), had <4 days of valid actigraphy data (*n*=1919, see below for the definition of a valid day), had missing average activity data for any hourly window (*n*=49) or had an unknown diabetes status (*n*=248). Thus the sample for the analysis focusing on diabetes status included 7074 participants. For the analyses focusing on the glycaemic biomarkers, which were measured in a randomly selected sample who visited the morning session at the NHANES mobile examination centre, we further excluded those with missing outcome data for the specific analysis and those taking diabetes medication, resulting in a sample size of 2820, 2769, 2769 and 2582 for fasting glucose, fasting insulin, HOMA-IR and OGTT, respectively.

### Physical activity measurements

The protocol for the physical activity monitor component in NHANES 2011–2014 is available online [22]. Briefly, participants aged ≥3 years wore a triaxial actigraph device (ActiGraph GT3X+) on the wrist of the non-dominant hand for seven full days (midnight to midnight) to record 24 h movements at a sampling frequency of 80 Hz. Raw data were processed to determine whether signal patterns were unlikely to be caused by human movement, and those determined as such were flagged. Details about data quality and the meaning of flags are published on the NHANES website [23]. Using a published open-source algorithm, John et al harmonised and aggregated raw accelerometry data in NHANES 2011–2014 to generate a monitor-independent movement summary (MIMS) unit, which was optimised to capture normal human motion [24]. Using aggregated data, activity at the minute level was further classified as ‘wake wear’, ‘sleep wear’ or ‘non-wear’ [24]. Using the same criteria as in our previous studies [25–27], we defined a valid minute as one labelled as wake or sleep, and a valid day as one having at least 20 h of valid data. This classification was used to exclude participants with insufficient data (as indicated above). To measure total activity levels for each hour, we used the minute-level MIMS data because this measure was commonly used in epidemiological studies to examine physical activity patterns and their relationships with various health outcomes in the USA [3, 28, 29]. We calculated the mean of minute-level MIMS units for each hourly window defined by two methods: (1) relative to the average sleep midpoint of each individual (e.g. 0–59 min after the midpoint, primary method; see below for sleep midpoint calculation); and (2) based on clock time (e.g. 00:01–01:00 hours, secondary method).

To calculate the average sleep midpoint, we first determined the average time of sleep onset and offset for each individual. For this calculation, we focused on sleep time between the period of 20:00–10:00 hours to minimise the influence of unusual/extreme sleep windows that may be severely misaligned with the circadian clock. To determine the sleep onset time, we calculated the percentage of sleep wear time within a rolling 30 min window, starting from 20:00 hours, and defined sleep onset as the first minute labelled as sleep wear within the first rolling window during which the sleep wear time was 15 min (50%) or higher. To determine sleep offset, we applied the same process backward, starting from 10:00 hours: the first window was 09:31–10:00 hours, and if there were fewer than 15 min labelled as sleep, the window was rolled 1 min backward to 09:30–09:59 hours. This process continued until 15 or more minutes of sleep time were recorded within a window. The individual’s overall sleep midpoint was then defined as the midpoint between the averaged sleep onset and offset time across the recording period. We further calculated the sleep midpoint on weekends by using sleep onset and offset time between 20:00 hours on Friday and 10:00 hours on Sunday. We used the weekend sleep midpoint as an indicator of chronotype because it is less influenced by work schedule and other external constraints and thus more likely to reflect the individual’s internal propensity to sleep at a certain time of the day.

### Diabetes mellitus and glycaemic markers

The primary outcome was diabetes status. Individuals with diabetes mellitus were defined using the following criteria: HbA_1c_ ≥48 mmol/mol (6.5%) as measured using a Tosoh automated glycohaemoglobin analyser HLC-723G8, or self-reported diagnosis. Secondary outcomes included four glycaemic markers (fasting glucose, fasting insulin, HOMA-IR and 2 h OGTT results). Details on laboratory assays for fasting glucose, fasting insulin, HOMA-IR and OGTT and for each survey cycle are published on the NHANES website [30–33]. We applied the conversion algorithm as recommended by NHANES to match the 2013–2014 fasting insulin values to the 2011–2012 values to account for differences in laboratory protocols between the cycles: insulin_2011–2012_ =$10^{\left(1.024\times{\text{log}}_{10}\left[{\text{insulin}}_{2013-2014}\right]-0.0802\right)}$. HOMA-IR was calculated as previously reported [34]. OGTT results were based on plasma glucose levels measured 2 h after ingesting 75 g glucose orally (Trutol glucose solution, Thermo Fisher Scientific). For the OGTT, there were several additional exclusion criteria, including haemophilia and chemotherapy safety exclusions, fasting for less than 9 h, taking insulin or oral medications for diabetes, refusing phlebotomy and not drinking the entire glucose solution within the allotted time. All four secondary outcome variables were log_*e*_-transformed to improve normality.

### Covariates

The following variables were included as covariates in regression analyses: sociodemographic characteristics and lifestyle factors (age, gender, race/ethnicity, education, household income, marital status, smoking, alcohol consumption; all as reported during home-based interviews), BMI (calculated based on weight and height measured at the NHANES mobile examination centre), total energy intake (estimated based on two 24 h dietary recalls), sleep duration (measured as the daily average of total minutes categorised as sleep wear), and total physical activity (measured as the average of the daily sum of per-minute MIMS values across the recording period).

### Statistical analysis

We divided hourly physical activity levels (mean MIMS as described above), defined either based on clock time or relative to the overall sleep midpoint, into quintiles, and used the lowest quintile as the reference group. For each 1 h window, we examined the association between quintiles of physical activity levels and diabetes status using multiple logistic regression, adjusted for age, gender, race/ethnicity, education, household income, marital status, smoking status, alcohol intake, total energy intake, sleep duration, sleep midpoint and total physical activity. For analyses focusing on glycaemic markers, we used multiple linear regression adjusted for the same covariates. *p* values for trend were determined by modelling quintiles of physical activity variables as continuous (i.e. 1–5 for Q1–Q5). For sensitivity analysis, we excluded participants with extreme sleep duration (≤4 or ≥10 h). We also performed subgroup analyses focusing on the primary outcome (i.e. diabetes) stratified by gender (men/women), age (<65 years/≥65 years), race/ethnicity (non-Hispanic white, non-Hispanic black, Hispanic), chronotype (weekend sleep midpoint before 03:15 (median) vs after 03:15 hours) and sleep duration (<7 h/≥7 h). For subgroup analyses, we modelled quintiles of hourly activity levels as a continuous variable to preserve statistical power, and relied on visualisation of the 24 h patterns of associations to identify potential similarities and differences in a qualitative fashion. To account for the complex, multi-stage probability sampling design of NHANES, we used the following: the full-sample mobile examination centre examination weight for the analysis focusing on diabetes, the fasting sub-sample weight for fasting glucose, fasting insulin and HOMA-IR, and the OGTT sub-sample weight for OGTT results. All analyses were performed using SAS 9.4 (SAS Institute).

## Results

Table 1 presents the study characteristics in the overall sample and by diabetes status. Compared to those without diabetes, participants with diabetes were older, had a lower education and household income, and exhibited a less healthy lifestyle, as evidenced by a higher prevalence of current smoking, obesity and lower total physical activity levels. However, people with diabetes were also less likely to report one or more alcohol drinks per day. The overall average midpoint of sleep was similar between the groups, but people with diabetes had a slightly earlier midpoint on weekends. Hourly physical activity levels relative to sleep midpoint for participants with and without diabetes are presented in ESM Fig. 2 and ESM Table 1, with the latter also showing the results for hourly windows defined by clock time. People with diabetes exhibited lower levels of physical activity during the day, but the overall 24 h patterns were generally similar, showing a plateau of higher activity levels between 7 and 17 h after the sleep midpoint, or between 10:01 and 19:00 hours.

**Table 1:** Patient characteristics by diabetes status: NHANES 2011–2014

	Total sample	Diabetes	
		No	Yes
*N*	7074	5751	1323
Age	49.7 (35.9–62.4)	47.3 (33.9–60.2)	61.5 (51.8–70.0)
Female	52.0	52.2	50.7
Race/ethnicity			
Non-Hispanic white	68.1	69.4	60.1
Non-Hispanic black	10.7	9.9	15.9
Hispanic	14.0	13.9	14.7
Other	7.1	6.8	9.3
Education less than high school	15.9	14.5	24.1
Household income <US$20k	14.8	13.7	21.7
Married	56.3	56.3	56.6
Current smoker	25.4	23.9	34.9
Alcohol, ≥1 drink/day	15.0	16.4	6.3
Living with obesity^a^	38.5	34.6	63.4
Total energy intake (kJ)			
Men	9774 (7653, 12,092)	9908 (7828, 12,205)	8841 (6548, 11,284)
Women	7263 (5870, 8970)	7347 (6004, 9159)	6673 (4954, 8217)
Sleep duration (h)			
<7	55.4	56.8	46.8
7–9	36.9	36.4	40.0
>9	7.7	6.9	13.2
Midpoint of sleep (24 h clock; HH:MM), median (IQR)			
Overall	02:56 (02:23–03:34)	02:56 (02:23–03:35)	02:52 (02:22–03:31)
Weekend^b^	03:11 (02:34–03:54)	03:13 (02:34–03:56)	03:02 (02:33–03:39)
Total physical activity, MIMS values/day (1000)	11.0 (9.0–13.2)	11.3 (9.4–13.4)	9.4 (7.6–11.2)

Values are percentages or median (IQR) and are weighted using sample weights

Diabetes defined as HbA_1c_ ≥48 mmol/mol (6.5%) or self-reported diagnosis of diabetes

^a^Defined as BMI ≥30 kg/m^2^

^b^Weekend sleep was defined as sleep on Friday and Saturday nights

In our sample, 18.7% (*n*=1323) of participants were classified as having diabetes. The associations between hourly physical activity levels and prevalent diabetes are presented in Fig. 1, Table 2 (hourly windows relative to sleep midpoint) and ESM Table 2 (hourly windows based on clock time). Overall, the temporal pattern of the associations was positive during the sleep window or around late night and early morning, and negative during wake time or in the late morning and afternoon. Specifically, during a 7 h window spanning from 2 h before the sleep midpoint to 5 h after, higher activity levels were associated with higher odds of diabetes, with a 36–103% increase when comparing the highest quintile with the lowest quintile (Fig. 1a). With an average sleep midpoint of 03:00 hours, this window largely overlapped the period of 00:01–07:00 hours in Fig. 1b. During the wake time/daytime, we observed a ‘double-dipping’ pattern (i.e. a pattern that exhibits two time windows during which higher activity was associated with lower odds of diabetes). The first window was centred around 9 h after the sleep midpoint (Fig. 1a) or around 11:01–12:00 hours clock time (Fig. 1b), where the highest quintile of activity was associated with 35% lower odds of diabetes (OR 0.65; 95% CI 0.44, 0.96). The second window was observed 12–17 h after the sleep midpoint (Fig. 1a), or around 14:01–19:00 hours (Fig. 1b), and it was both deeper and wider than the first window. During this time window, the highest quintiles were associated with 56% and 36% lower odds (OR 0.44; 95% CI 0.29, 0.69 and OR 0.64; 95% CI 0.43, 0.95 for 13 and 17 h after the sleep midpoint, respectively) . Excluding participants with extreme sleep duration had a limited impact on the study findings (changes in effect size <2% in all cases). Because the results were qualitatively similar regardless of which method we used to define the hourly windows, we focus on results using hourly windows relative to sleep midpoint for the remainder of the data presentation for better control of individual differences in sleep timing.

**Table 2:** Associations between average hourly physical activity and diabetes among NHANES (2011–2014) participants

Hour after sleep midpoint^a^	Diabetes, OR (95% CI), by quintiles of hourly physical activity					
	Q1	Q2	Q3	Q4	Q5	*p*_trend_
1	Ref	1.26 (0.92, 1.72)	1.58 (1.18, 2.11)	1.89 (1.46, 2.43)	1.71 (1.28, 2.30)	<0.0001
2	Ref	1.18 (0.90, 1.56)	1.36 (1.09, 1.70)	1.48 (1.12, 1.94)	1.61 (1.33, 1.96)	<0.0001
3	Ref	1.43 (1.03, 1.99)	1.38 (1.04, 1.83)	1.73 (1.40, 2.13)	1.76 (1.39, 2.23)	<0.0001
4	Ref	1.26 (1.01, 1.58)	1.86 (1.36, 2.55)	1.83 (1.31, 2.56)	2.03 (1.48, 2.79)	<0.0001
5	Ref	1.19 (0.90, 1.58)	1.52 (1.19, 1.94)	1.83 (1.38, 2.42)	1.41 (0.94, 2.10)	0.001
6	Ref	1.39 (1.13, 1.72)	1.44 (1.10, 1.89)	1.21 (0.92, 1.60)	1.04 (0.72, 1.49)	0.89
7	Ref	1.37 (1.07, 1.75)	1.13 (0.88, 1.46)	0.93 (0.7, 1.23)	0.88 (0.66, 1.18)	0.06
8	Ref	1.08 (0.86, 1.36)	0.95 (0.76, 1.20)	0.84 (0.57, 1.25)	0.70 (0.48, 1.04)	0.07
9	Ref	1.25 (0.92, 1.69)	0.80 (0.57, 1.14)	0.82 (0.56, 1.21)	0.65 (0.44, 0.96)	0.01
10	Ref	0.81 (0.60, 1.09)	0.95 (0.66, 1.36)	0.75 (0.53, 1.06)	0.65 (0.41, 1.03)	0.10
11	Ref	1.15 (0.90, 1.48)	1.07 (0.79, 1.46)	1.09 (0.81, 1.47)	0.92 (0.61, 1.39)	0.80
12	Ref	1.05 (0.81, 1.36)	0.84 (0.57, 1.26)	0.84 (0.56, 1.26)	0.51 (0.35, 0.75)	0.01
13	Ref	0.93 (0.75, 1.16)	0.65 (0.48, 0.87)	0.71 (0.46, 1.11)	0.44 (0.29, 0.69)	0.003
14	Ref	0.88 (0.70, 1.11)	0.80 (0.55, 1.16)	0.63 (0.45, 0.88)	0.44 (0.31, 0.63)	<0.0001
15	Ref	0.91 (0.62, 1.32)	0.79 (0.54, 1.14)	0.56 (0.35, 0.91)	0.48 (0.28, 0.83)	0.001
16	Ref	0.87 (0.65, 1.18)	0.78 (0.60, 1.02)	0.61 (0.43, 0.86)	0.55 (0.32, 0.97)	0.01
17	Ref	0.98 (0.82, 1.17)	0.94 (0.68, 1.31)	0.79 (0.59, 1.06)	0.64 (0.43, 0.95)	0.04
18	Ref	1.23 (0.90, 1.69)	1.44 (0.99, 2.09)	1.32 (0.81, 2.16)	1.03 (0.58, 1.84)	0.61
19	Ref	1.23 (0.86, 1.76)	1.27 (0.99, 1.63)	1.05 (0.80, 1.37)	1.22 (0.79, 1.87)	0.55
20	Ref	0.95 (0.68, 1.32)	1.12 (0.93, 1.35)	1.02 (0.73, 1.42)	1.04 (0.73, 1.49)	0.66
21	Ref	0.92 (0.66, 1.30)	1.02 (0.75, 1.40)	1.23 (0.91, 1.67)	1.02 (0.69, 1.51)	0.34
22	Ref	0.91 (0.69, 1.19)	0.89 (0.70, 1.14)	1.14 (0.83, 1.56)	1.25 (0.90, 1.73)	0.15
23	Ref	1.18 (0.92, 1.51)	1.13 (0.92, 1.38)	1.53 (1.17, 2.00)	1.36 (1.10, 1.69)	0.001
24	Ref	1.46 (1.10, 1.93)	1.45 (1.03, 2.04)	1.98 (1.49, 2.63)	1.89 (1.40, 2.55)	<0.0001

Risk is expressed as OR (95% CI) for prevalent diabetes by quintiles of physical activity. For example, in the first data row, individuals who are highly active within 1 h after sleep midpoint (in the highest quartile [Q5] of the distribution) have 1.71-fold higher odds of having diabetes when compared with people who are least active at that time (in Q1 of the distribution). The OR are derived from multiple logistic regression models adjusted for age (continuous) and gender (men/women), race/ethnicity (non-Hispanic white, non-Hispanic black, Hispanic, others), education (less than high school, high school graduate, some college, college graduate or above), household income (<US$20k, US$20k–44.9k, US$45k–74.9k, ≥US$75k), marital status (married/not married), smoking (current smoker, former smoker, never smoker or fewer than 100 cigarettes in life), alcohol consumption (<1 drink/week, 1 drink/week – <1 drink/day, ≥1 drink/day), BMI (kg/m^2^: <18.5, 18.5 – <25, 25 – <30, ≥30), total energy intake (continuous), sleep duration (<7, 7–9, >9 h), sleep midpoint (continuous) and daily total physical activity (continuous)

Diabetes was defined as HbA_1c_ ≥48 mmol/mol (6.5%) or self-reported diagnosis of diabetes

^a^Each window is labelled as the number of hours passed beyond sleep midpoint at the end of the hour. For example, the first hourly window started after the sleep midpoint and ended 60 min after that

**Fig. 2:**
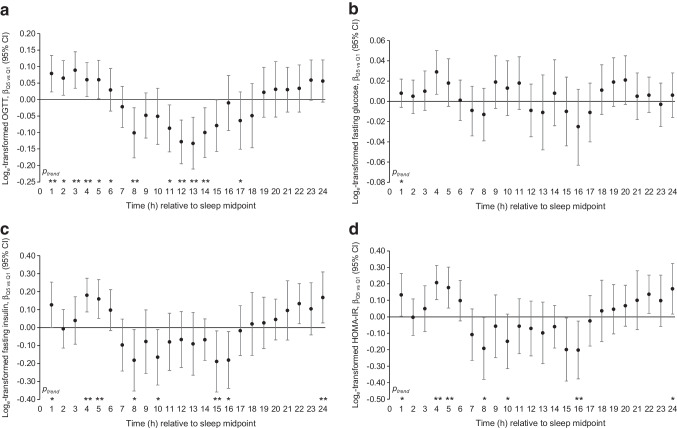
Associations between average hourly physical activity and 2 h OGTT glucose (**a**), fasting glucose (**b**), fasting insulin (**c**) and HOMA-IR (**d**) among NHANES (2011–2014) participants, comparing the highest quintile of hourly activity with the lowest quintile. Each hourly window was determined as relative to the average sleep midpoint of the individual (e.g. the first hourly window started after the sleep midpoint and ended 60 min after that). Associations are expressed as the β coefficient and 95% CI, comparing individuals in the highest activity quintile for each hourly window (Q5) with those in the lowest quintile (Q1), derived from multiple logistic regression models adjusted for age (continuous) and gender (men/women), race/ethnicity (non-Hispanic white, non-Hispanic black, Hispanic, others), education (less than high school, high school graduate, some college, college graduate or above), household income (<US$20k, US$20k–44.9k, US$45k–74.9k, ≥US$75k), marital status (married/not married), smoking (current smoker, former smoker, never smoker or fewer than 100 cigarettes in life), alcohol consumption (<1 drink/week, 1 drink/week but – <1 drink/day, ≥1 drink/day), BMI (kg/m^2^: <18.5, 18.5 – <25, 25 – <30, ≥30), total energy intake (continuous), sleep duration (<7, 7–9, >9 h), sleep midpoint (continuous) and daily total physical activity (continuous). *p*_trend_ represents the *p* values for trend, with hourly physical activity modelled as a continuous variable (^*^p<0.05; ^**^p<0.01)

The results for glycaemic markers are presented in Fig. 2 and ESM Tables 3–6. The pattern of associations of hourly activity levels with 2 h glucose results during OGTT (Fig. 2a) is similar to that with prevalent diabetes, showing positive relationships linked to higher levels of activity around the nightly sleep window, and double-dipping negative relationships during wake time. However, we did not observe any associations between hourly physical activity levels and fasting glucose (Fig. 2b), and the associations with fasting insulin (Fig. 2c) and HOMA-IR (Fig. 2d) were also weaker than those observed for diabetes and 2 h OGTT results.

The results from subgroup analyses by age, gender, race/ethnicity, chronotype and sleep duration are presented in ESM Figures 3–7 and ESM Tables 7–11. Overall, we did not detect meaningful differences in results across the investigated subgroups. However, the night-time/sleep-time window during which higher activity levels were positively associated with prevalent diabetes appeared to be somewhat wider among men and individuals who were younger than 65 years or who had a sleep duration <7 h. Moreover, in subgroup analyses by race/ethnicity, the double-dipping pattern of associations between activity and prevalent diabetes was observed in non-Hispanic black and white groups, but not in the Hispanic group, with the latter showing a single period of negative associations between 9–13 h after the sleep midpoint. Finally, in analyses stratified by chronotype or weekend sleep midpoint, both the positive associations during sleep time and the negative associations during wake time appeared stronger among people with a later chronotype.

## Discussion

In our nationally representative sample of US adults, we observed that higher activity levels during the wake window and during daytime were associated with lower diabetes risk. Notably, these associations were observed after controlling for conventional aspects of human rest–activity behaviours, including total physical activity, sleep duration and sleep timing, suggesting that these results reflect a unique temporal pattern in the association between physical activity and metabolic health. In contrast, greater physical activity levels during night-time were associated with higher diabetes risk. The results in various demographic subgroups and by chronotype were generally consistent.

In humans, insulin resistance/sensitivity is in part regulated by the circadian system, and exhibits a clear diurnal pattern, with higher insulin sensitivity in the morning and lower sensitivity later in the day and at night [35, 36]. This has led to the hypothesis that the metabolic effect of exercise/physical activity on glycaemic management may also fluctuate during the 24 h cycle [37], and this hypothesis is supported by a number of observational and randomised intervention studies [7–14]. Several of these studies, including both observational and randomised intervention studies using various analytical strategies, found that afternoon physical activity may be more beneficial than morning activity for insulin resistance [7, 10, 12, 14]. However, not all studies support the hypothesis of a more favourable role of afternoon physical activity over activity at other times during the day. In a cohort study on participants in the UK Biobank, both morning (06:00–12:00 hours) and afternoon (12:00–18:00 hours) physical activity levels were associated with lower diabetes risk, with similar risk estimates [13]. A number of randomised trials also found no differences for multiple cardiometabolic outcomes [8, 11]. An earlier analysis in the Look AHEAD trial reported that morning bout-related moderate-to-vigorous-intensity physical activity was associated with higher cardiorespiratory fitness, and, counterintuitively, with a higher cardiovascular risk as measured by the Framingham risk score. These associations were only observed in men, not in women [9]. Finally, it has been found that higher activity in the early morning (04:00–09:00 hours) was associated with lower fasting glucose, fasting insulin and HOMA-IR. In the same study, afternoon activity was not associated with markers of glycaemic management, but was inversely associated with BMI [15]. Thus, epidemiological evidence has not been consistent in demonstrating clear timing effects of physical activity on measures reflecting glucose homeostasis.

We believe that our study may offer important clues for the inconsistent findings in previous research. First, our findings suggested that both morning and afternoon physical activity may be associated with better metabolic outcomes, but the specific time windows during which such an effect was observed were relatively short. This was particularly true for the morning, which showed a brief 1–2 h window with moderate effect size. Thus, the inconsistent findings in previous studies may be partially attributable to their varied definition of time windows, as well as individual differences in circadian timing, which we examined by defining time windows based on clock time as well as the midpoint time of sleep. Second, although the results were similar in stratified analyses, we observed some evidence suggesting that the temporal patterns of the activity/diabetes relationship may vary across demographic groups and by chronotype. Heterogeneity in the study sample characteristics in previous studies may have contributed to the heterogeneity in study findings. Third, our findings also suggest that the strength of the results may differ across different study outcomes. For example, the temporal patterns appeared to be stronger for diabetes status, which was assessed largely based on HbA_1c_ results, and 2 h glucose OGTT results, but weak to null for the other three glycaemic markers. Mechanistic studies have revealed complex circadian regulation of different aspects of metabolism [38]. The previous studies examined a wide range of cardiometabolic outcomes, which may have contributed to the mixed findings. Finally, the various studies also focused on different types and/or intensity levels of physical activity, which may have different metabolic effects.

We observed a positive association between higher activity levels during the sleep window/night-time and diabetes, which is consistent with the well-established adverse metabolic effects of sleep deficiency [39]. Higher mean levels of physical activity during the night may be caused by a multitude of factors, including insufficient sleep duration, poor sleep maintenance, higher sleep fragmentation and/or irregular sleep timing, all of which have been consistently linked with diabetes [4, 40, 41]. Therefore, our results using hourly activity windows over the 24 h rest–activity cycle showcase the potential of temporally granular analysis in identifying subtle differences in the metabolic effects of rest and activity.

In addition to the use of novel approaches providing novel insights, the present study also had a number of limitations that warrant consideration when interpreting the study results. First, NHANES is a cross-sectional study, and the inherent temporal ambiguity makes it challenging to establish cause and effect. Future studies should take advantage of longitudinal data to elucidate the nature of the relationship between physical activity timing and diabetes. Second, we used sleep timing as a proxy measure of internal circadian timing, and weekend sleep timing as a measure of chronotype. However, it is well established that sleep is regulated by both circadian rhythm and the homeostatic process, and is heavily influenced by environmental factors. To accurately assess circadian timing requires measurements such as dim-light melatonin onset and core body temperature rhythm. Unfortunately, such information was not available in NHANES. Third, the temporal pattern of physical activity of an individual is determined by many external constraints, such as work schedule, domestic obligations, access to exercise facilities, and social interactions, and these factors are probably correlated with many sociodemographic, socioeconomic and cultural factors that may also influence metabolic health. Although we controlled for a number of covariates in the model, not all factors were measures in NHANES. In particular, we did not have information on nightshift work, which can cause major alterations in 24 h physical activity patterns, and has been linked to diabetes previously [42]. However, by determining sleep timing during the 20:00–10:00 hours time window, we essentially excluded those with extreme sleep schedules. It is worth noting that only 0.5% of the total days recorded in the overall sample were excluded according to this criterion, suggesting that there were few people with an unconventional sleep pattern. Future studies with a larger sample of shift workers are needed to assess the relationship between physical activity timing and metabolic health in this high-risk population. Fourth, the timing of physical activity relative to meal timing (e.g. before or after meals) may also modify its short-term and long-term effects on glycaemic management. Unfortunately, we do not have meal timing information on the days when actigraphy data were collected. Future studies should investigate how the interaction between exercise timing and meal timing influences the metabolic effect of physical activity. Fifth, our study focused on total physical activity and did not differentiate among intensity levels. Previous studies have suggested that light activity and moderate-to-vigorous physical activity may have different physiological effects, with the latter providing greater metabolic benefits [43]. Similarly, the health effects of physical activity may also differ by type (e.g. aerobic vs resistance training) [44]. Therefore, future studies should assess the potential modifying effect of timing for specific physical activity types and intensity levels. Finally, as in any observational study, residual confounding is always a concern.

In conclusion, our study contributes to a growing body of literature highlighting the important role of timing of physical activity in metabolic health. If confirmed by prospective and randomised trials, the findings suggest that prescribing daytime physical activity or exercise during particular time windows and improving sleep at night may improve glycaemic management and reduce diabetes risk. Moreover, the results may also help in developing digital biomarkers based on data from wearable devices for use in diabetes risk prediction and disease management in the clinical setting.

## Supplementary Information

Below is the link to the electronic supplementary material.

ESM (PDF 742 KB)

## Data Availability

All datasets used in this analysis are publicly available from the NHANES website (https://wwwn.cdc.gov/nchs/nhanes/).

## Abbreviations

MIMS: Monitor-independent movement summary
NHANES: National Health and Nutrition Examination Survey
