# Supplementary material for: Associations between the timing of 24 h physical activity and diabetes mellitus: results from a nationally representative sample of the US population
Source: Diabetologia. 2025 Feb 21;68(5):1005–15. doi: 10.1007/s00125-025-06368-9 (PMC12021934; doi:10.1007/s00125-025-06368-9)
Supplement: Supplementary file 1 — ESM (PDF 742 KB) [file 125_2025_6368_MOESM1_ESM.pdf]

ESM Table 1. Hourly average physical activity (count/minute, median (IQR)) patterns of NHANES participants with and without diabetes.

Hourly window	Hourly physical activity (count/minute, median (IQR)) by diabetes status		Hourly physical activity (count/minute, median (IQR)) by diabetes status	
	**No**	**Yes**	**No**	**Yes**
	**Hourly windows defined by clock time**		**Hourly windows relative to sleep midpoint**	
0:00-0:59	2.03 (0.88z-4.62)	1.88 (0.87-3.97)	0.80 (0.51-1.41)	0.97 (0.58-1.64)
1:00-1:59	1.17 (0.63-2.70)	1.36 (0.77-2.41)	0.86 (0.55-1.53)	1.01 (0.65-1.86)
2:00-2:59	0.94 (0.58-1.94)	1.11 (0.69-1.99)	1.28 (0.71-2.72)	1.43 (0.85-3.01)
3:00-3:59	0.89 (0.56-1.67)	1.10 (0.70-1.86)	4.20 (1.85-8.13)	3.72 (1.71-6.38)
4:00-4:59	1.00 (0.62-1.94)	1.26 (0.72-2.17)	9.49 (5.94-12.98)	7.71 (4.82-10.72)
5:00-5:59	1.55 (0.78-4.08)	1.84 (0.91-4.05)	12.06 (8.78-15.62)	10.14 (7.64-13.45)
6:00-6:59	4.63 (1.70-9.61)	3.89 (1.62-8.24)	13.39 (10.40-16.94)	11.45 (8.83-14.59)
7:00-7:59	9.39 (4.86-13.54)	7.73 (4.18-11.19)	13.85 (10.99-17.34)	11.87 (9.23-14.84)
8:00-8:59	11.70 (7.68-15.48)	9.70 (6.91-13.42)	14.03 (11.26-17.43)	12.02 (9.31-14.94)
9:00-9:59	12.65 (9.32-16.47)	11.13 (8.01-14.13)	14.12 (11.42-17.48)	12.18 (9.42-15.08)
10:00-10:59	13.14 (10.14-16.91)	11.21 (8.75-14.6)	13.89 (11.19-17.14)	11.58 (9.12-14.47)
11:00-11:59	13.64 (10.68-17.09)	11.65 (8.97-14.63)	13.75 (10.85-16.98)	11.07 (8.58-14.23)
12:00-12:59	13.89 (11.22-17.03)	11.87 (9.36-14.53)	13.68 (10.79-16.70)	11.12 (8.43-13.75)
13:00-13:59	13.71 (10.82-17.00)	11.42 (8.87-14.06)	13.66 (10.97-16.72)	11.11 (8.45-13.69)
14:00-14:59	13.37 (10.61-16.64)	10.87 (8.48-13.72)	13.95 (11.18-16.87)	11.02 (8.62-13.93)
15:00-15:59	13.32 (10.56-16.49)	10.81 (8.2-13.77)	13.84 (11.01-16.85)	11.03 (8.39-13.82)
16:00-16:59	13.46 (10.71-16.44)	10.75 (8.24-13.56)	13.04 (10.16-16.03)	10.60 (7.82-13.33)
17:00-17:59	13.80 (10.95-16.86)	10.84 (8.45-13.74)	11.85 (9.07-14.91)	9.54 (7.08-12.08)
18:00-18:59	13.64 (10.68-16.61)	10.7 (8.22-13.57)	10.17 (7.63-12.97)	8.21 (5.95-10.78)
19:00-19:59	12.61 (9.76-15.75)	9.87 (7.39-12.90)	7.78 (5.54-10.20)	6.35 (4.38-9.03)
20:00-20:59	11.36 (8.19-14.51)	8.97 (6.58-11.55)	4.31 (2.65-6.58)	3.75 (2.07-6.08)
21:00-21:59	9.44 (6.70-12.71)	7.45 (5.01-10.45)	1.89 (0.93-3.67)	1.91 (0.91-3.61)
22:00-22:59	7.27 (4.40-10.54)	5.91 (3.36-8.91)	1.03 (0.57-2.11)	1.22 (0.69-2.28)
23:00-23:59	4.26 (1.84-7.72)	3.64 (1.67-6.62)	0.81 (0.51-1.57)	1.04 (0.64-1.75)

Abbreviations: IQR, interquartile range; NHANES, National Health and Nutrition Examination Survey.

ESM Table 2. Associations[a] between average hourly physical activity and prevalent diabetes[b] among NHANES (2011-2014) participants.

Hourly windows defined by clock time	Diabetes, OR (95% CI), by quintiles of hourly physical activity					
	Q1	Q2	Q3	Q4	Q5	p_{trend}
0:01-1:00	ref	1.26 (0.87, 1.81)	1.41 (1.02, 1.93)	1.58 (1.13, 2.20)	1.70 (1.22, 2.37)	*0.001*
1:01-2:00	ref	0.97 (0.75, 1.27)	1.14 (0.87, 1.50)	1.46 (1.11, 1.93)	1.33 (0.93, 1.90)	*0.02*
2:01-3:00	ref	1.24 (0.91, 1.70)	1.69 (1.29, 2.21)	1.86 (1.42, 2.43)	1.76 (1.21, 2.57)	*<.0001*
3:01-4:00	ref	1.05 (0.77, 1.44)	1.69 (1.37, 2.09)	1.72 (1.39, 2.14)	1.48 (1.15, 1.91)	*<.0001*
4:01-5:00	ref	1.36 (1.00, 1.83)	1.75 (1.36, 2.27)	1.82 (1.40, 2.36)	1.60 (1.26, 2.02)	*<.0001*
5:01-6:00	ref	1.21 (0.90, 1.62)	1.52 (1.11, 2.09)	1.70 (1.36, 2.11)	1.76 (1.25, 2.49)	*<.0001*
6:01-7:00	ref	1.26 (1.02, 1.57)	1.46 (1.10, 1.93)	2.04 (1.63, 2.55)	1.73 (1.34, 2.24)	*<.0001*
7:01-8:00	ref	1.09 (0.80, 1.47)	1.34 (0.96, 1.88)	1.26 (0.91, 1.74)	1.32 (0.83, 2.09)	*0.16*
8:01-9:00	ref	1.32 (0.93, 1.86)	1.28 (0.92, 1.78)	0.99 (0.64, 1.51)	1.02 (0.72, 1.45)	*0.45*
9:01-10:00	ref	1.16 (0.91, 1.48)	1.02 (0.75, 1.40)	0.94 (0.62, 1.42)	0.65 (0.44, 0.98)	*0.05*
10:01-11:00	ref	1.20 (0.86, 1.67)	1.16 (0.85, 1.59)	0.98 (0.72, 1.32)	0.89 (0.61, 1.31)	*0.27*
11:01-12:00	ref	1.22 (0.97, 1.54)	0.95 (0.71, 1.27)	0.87 (0.64, 1.19)	0.77 (0.57, 1.04)	*0.01*
12:01-13:00	ref	0.90 (0.69, 1.19)	0.97 (0.73, 1.30)	0.84 (0.56, 1.26)	0.66 (0.43, 1.01)	*0.12*
13:01-14:00	ref	1.20 (0.93, 1.56)	1.10 (0.78, 1.56)	1.10 (0.78, 1.56)	0.71 (0.48, 1.06)	*0.29*
14:01-15:00	ref	1.14 (0.92, 1.41)	0.91 (0.68, 1.22)	0.83 (0.59, 1.18)	0.53 (0.36, 0.79)	*0.003*
15:01-16:00	ref	1.23 (0.98, 1.54)	0.76 (0.52, 1.10)	0.74 (0.52, 1.06)	0.52 (0.33, 0.80)	*0.001*
16:01-17:00	ref	0.77 (0.56, 1.06)	0.72 (0.49, 1.06)	0.73 (0.48, 1.10)	0.49 (0.30, 0.78)	*0.02*
17:01-18:00	ref	0.71 (0.46, 1.11)	0.72 (0.52, 0.98)	0.55 (0.34, 0.89)	0.44 (0.24, 0.83)	*0.01*
18:01-19:00	ref	0.85 (0.66, 1.10)	0.72 (0.55, 0.95)	0.70 (0.48, 1.02)	0.38 (0.25, 0.60)	*0.0002*
19:01-20:00	ref	1.18 (0.92, 1.51)	0.99 (0.79, 1.23)	0.86 (0.62, 1.20)	0.87 (0.54, 1.40)	*0.24*
20:01-21:00	ref	1.23 (0.92, 1.64)	1.12 (0.79, 1.57)	1.16 (0.77, 1.74)	1.03 (0.66, 1.60)	*0.93*
21:01-22:00	ref	1.32 (0.99, 1.74)	1.70 (1.27, 2.29)	1.12 (0.81, 1.55)	1.19 (0.77, 1.83)	*0.47*
22:01-23:00	ref	0.98 (0.78, 1.23)	0.99 (0.77, 1.29)	1.06 (0.79, 1.41)	1.04 (0.71, 1.52)	*0.69*
23:01-24:00	ref	0.95 (0.68, 1.33)	1.12 (0.79, 1.58)	1.35 (0.95, 1.92)	1.13 (0.77, 1.67)	*0.07*

[a] Expressed as OR (95% CI) for prevalent diabetes. For example, in the first data row, individuals who are highly active within 1 hour after sleep midpoint (in the highest quartile (Q5) of the distribution) have 1.71-fold higher odds of having diabetes when compared to people who are least active at that time (in Q1 of the distribution). OR are derived from multiple logistic regression models adjusted for age, sex, race/ethnicity, education, household income, marital status, smoking status, alcohol intake, total energy intake, sleep duration, sleep midpoint and total physical activity.

[b] Defined as $HbA_{1c} \geq 6.5\%$ or self-reported diagnosis of diabetes.

Abbreviations: CI, confidence interval; HbA_{1c}, haemoglobin A1c; NHANES, National Health and Nutrition Examination Survey; OR, odds ratio; Q, quintile.

ESM Table 3 Associations[a] between average hourly physical activity and 2-hour glucose during OGTT among NHANES (2011-2014) participants.

**Hourly windows relative to sleep midpoint**	**Log-transformed 2-hour glucose during OGTT (β (95% CI)), by quintiles of hourly physical activity**					
	**Q1**	**Q2**	**Q3**	**Q4**	**Q5**	p_{trend}
0:00-0:59	ref	0.016 (-0.034, 0.065)	0.057 (0.023, 0.092)	0.061 (-0.006, 0.128)	0.079 (0.023, 0.134)	*0.002*
1:00-1:59	ref	0.028 (-0.014, 0.070)	-0.005 (-0.059, 0.049)	0.059 (0, 0.118)	0.065 (0.013, 0.118)	*0.02*
2:00-2:59	ref	-0.010 (-0.074, 0.054)	0.034 (-0.021, 0.089)	0.039 (-0.01, 0.089)	0.089 (0.034, 0.145)	*0.001*
3:00-3:59	ref	0.001 (-0.038, 0.040)	0.065 (0.001, 0.129)	0.053 (-0.001, 0.107)	0.060 (0.009, 0.112)	*0.01*
4:00-4:59	ref	0.003 (-0.034, 0.040)	0.065 (0.020, 0.110)	0.014 (-0.025, 0.052)	0.060 (0.002, 0.119)	*0.04*
5:00-5:59	ref	-0.060 (-0.104, -0.015)	-0.008 (-0.069, 0.052)	0.024 (-0.049, 0.096)	0.029 (-0.035, 0.094)	*0.04*
6:00-6:59	ref	-0.047 (-0.095, 0.001)	-0.023 (-0.076, 0.030)	-0.019 (-0.078, 0.040)	-0.022 (-0.085, 0.040)	*0.97*
7:00-7:59	ref	-0.040 (-0.095, 0.015)	-0.037 (-0.096, 0.022)	-0.087 (-0.150, -0.023)	-0.101 (-0.177, -0.025)	*0.01*
8:00-8:59	ref	0.001 (-0.055, 0.056)	-0.045 (-0.100, 0.009)	-0.021 (-0.077, 0.036)	-0.048 (-0.117, 0.020)	*0.15*
9:00-9:59	ref	-0.040 (-0.107, 0.027)	-0.021 (-0.104, 0.061)	-0.023 (-0.104, 0.059)	-0.051 (-0.136, 0.034)	*0.47*
10:00-10:59	ref	-0.036 (-0.091, 0.018)	-0.030 (-0.098, 0.037)	-0.082 (-0.144, -0.020)	-0.087 (-0.159, -0.016)	*0.02*
11:00-11:59	ref	-0.035 (-0.090, 0.021)	-0.064 (-0.112, -0.017)	-0.101 (-0.166, -0.036)	-0.128 (-0.195, -0.062)	*0.0002*
12:00-12:59	ref	-0.049 (-0.108, 0.010)	-0.077 (-0.138, -0.015)	-0.099 (-0.161, -0.037)	-0.133 (-0.211, -0.054)	*0.001*
13:00-13:59	ref	-0.048 (-0.107, 0.011)	-0.046 (-0.100, 0.007)	-0.089 (-0.169, -0.010)	-0.100 (-0.176, -0.025)	*0.01*
14:00-14:59	ref	-0.030 (-0.097, 0.037)	-0.032 (-0.099, 0.036)	-0.039 (-0.108, 0.029)	-0.079 (-0.158, -0.001)	*0.09*
15:00-15:59	ref	-0.001 (-0.066, 0.064)	0.032 (-0.028, 0.091)	-0.024 (-0.097, 0.050)	-0.010 (-0.094, 0.073)	*0.56*
16:00-16:59	ref	-0.005 (-0.072, 0.062)	-0.049 (-0.118, 0.021)	-0.068 (-0.138, 0.002)	-0.064 (-0.151, 0.023)	*0.05*
17:00-17:59	ref	-0.044 (-0.093, 0.005)	-0.048 (-0.116, 0.020)	-0.047 (-0.130, 0.035)	-0.049 (-0.146, 0.048)	*0.46*
18:00-18:59	ref	0.011 (-0.061, 0.083)	-0.002 (-0.057, 0.054)	0.020 (-0.046, 0.087)	0.022 (-0.054, 0.098)	*0.49*
19:00-19:59	ref	0.013 (-0.045, 0.072)	0.018 (-0.044, 0.08)	-0.006 (-0.078, 0.066)	0.031 (-0.053, 0.115)	*0.68*
20:00-20:59	ref	-0.017 (-0.085, 0.050)	0.017 (-0.053, 0.088)	-0.011 (-0.085, 0.064)	0.030 (-0.038, 0.098)	*0.31*
21:00-21:59	ref	-0.016 (-0.078, 0.046)	-0.018 (-0.075, 0.040)	-0.012 (-0.059, 0.035)	0.034 (-0.038, 0.105)	*0.38*
22:00-22:59	ref	0.033 (-0.026, 0.093)	-0.024 (-0.076, 0.028)	-0.004 (-0.061, 0.053)	0.059 (-0.002, 0.120)	*0.40*
23:00-23:59	ref	0.007 (-0.061, 0.075)	-0.009 (-0.073, 0.054)	0.034 (-0.027, 0.095)	0.056 (-0.008, 0.120)	*0.07*

[a] Expressed as beta coefficient and 95% CI derived from multiple linear regression models adjusted for age, sex, race/ethnicity, education, household income, marital status, smoking status, alcohol intake, total energy intake, sleep duration, sleep midpoint and total physical activity.
Abbreviations: β, beta coefficient; CI, confidence interval; NHANES, National Health and Nutrition Examination Survey; OGTT, oral glucose tolerance test; Q, quintile.

ESM Table 4 Associations [a] between average hourly physical activity and fasting glucose among NHANES (2011-2014) participants.

Hourly windows relative to sleep midpoint	Log-transformed fasting glucose (β (95% CI)), by quintiles of hourly physical activity					
	**Q1**	**Q2**	**Q3**	**Q4**	**Q5**	p_{trend}
0:00-0:59	ref	0.013 (-0.008, 0.034)	0.021 (0.006, 0.036)	0.022 (0.003, 0.040)	0.008 (-0.006, 0.022)	*0.03*
1:00-1:59	ref	-0.010 (-0.024, 0.003)	0.017 (-0.003, 0.037)	0.015 (-0.004, 0.034)	0.005 (-0.012, 0.021)	*0.07*
2:00-2:59	ref	-0.001 (-0.024, 0.022)	0 (-0.019, 0.019)	0.013 (-0.002, 0.029)	0.010 (-0.009, 0.030)	*0.07*
3:00-3:59	ref	0.029 (0.009, 0.049)	0.023 (-0.002, 0.047)	0.017 (0, 0.034)	0.029 (0.007, 0.050)	*0.04*
4:00-4:59	ref	0.002 (-0.015, 0.019)	0.019 (0.004, 0.034)	0.010 (-0.014, 0.033)	0.018 (-0.005, 0.042)	*0.12*
5:00-5:59	ref	0.008 (-0.010, 0.027)	0.014 (-0.004, 0.031)	0.002 (-0.018, 0.022)	0.001 (-0.019, 0.021)	*0.76*
6:00-6:59	ref	0.013 (-0.016, 0.041)	0.002 (-0.027, 0.032)	0.002 (-0.03, 0.034)	-0.009 (-0.034, 0.015)	*0.24*
7:00-7:59	ref	0.006 (-0.017, 0.028)	0 (-0.026, 0.027)	-0.007 (-0.031, 0.018)	-0.013 (-0.039, 0.013)	*0.11*
8:00-8:59	ref	0.017 (0.001, 0.033)	0.004 (-0.012, 0.021)	0.015 (-0.003, 0.032)	0.019 (-0.007, 0.045)	*0.23*
9:00-9:59	ref	0.016 (-0.014, 0.046)	0.012 (-0.011, 0.036)	0.012 (-0.014, 0.038)	0.013 (-0.014, 0.040)	*0.55*
10:00-10:59	ref	0.018 (-0.004, 0.040)	0.022 (0.002, 0.043)	0.013 (-0.009, 0.035)	0.018 (-0.008, 0.044)	*0.52*
11:00-11:59	ref	0.008 (-0.017, 0.033)	-0.003 (-0.03, 0.024)	-0.011 (-0.039, 0.017)	-0.009 (-0.035, 0.017)	*0.19*
12:00-12:59	ref	-0.002 (-0.031, 0.027)	-0.021 (-0.05, 0.008)	-0.011 (-0.038, 0.015)	-0.011 (-0.048, 0.026)	*0.41*
13:00-13:59	ref	0.005 (-0.024, 0.034)	-0.009 (-0.034, 0.016)	-0.001 (-0.03, 0.027)	0.008 (-0.024, 0.041)	*0.81*
14:00-14:59	ref	-0.007 (-0.033, 0.019)	0.009 (-0.023, 0.041)	-0.007 (-0.037, 0.023)	-0.010 (-0.044, 0.024)	*0.52*
15:00-15:59	ref	-0.012 (-0.042, 0.017)	-0.016 (-0.048, 0.016)	-0.032 (-0.068, 0.004)	-0.025 (-0.063, 0.012)	*0.09*
16:00-16:59	ref	0.012 (-0.010, 0.033)	-0.012 (-0.034, 0.010)	-0.002 (-0.027, 0.023)	-0.011 (-0.040, 0.018)	*0.29*
17:00-17:59	ref	0.014 (-0.003, 0.031)	0.026 (0.005, 0.047)	0.023 (0, 0.045)	0.011 (-0.013, 0.036)	*0.44*
18:00-18:59	ref	0.018 (-0.006, 0.043)	0.010 (-0.010, 0.029)	0.021 (0.004, 0.039)	0.019 (-0.005, 0.043)	*0.24*
19:00-19:59	ref	0.004 (-0.015, 0.024)	0.011 (-0.006, 0.027)	0.004 (-0.017, 0.025)	0.021 (-0.003, 0.045)	*0.15*
20:00-20:59	ref	0.006 (-0.017, 0.030)	0.013 (-0.015, 0.040)	0.002 (-0.023, 0.027)	0.005 (-0.018, 0.028)	*0.93*
21:00-21:59	ref	0.011 (-0.015, 0.038)	0.001 (-0.016, 0.019)	0.007 (-0.011, 0.026)	0.006 (-0.011, 0.024)	*0.67*
22:00-22:59	ref	-0.008 (-0.030, 0.014)	-0.014 (-0.032, 0.004)	0.009 (-0.011, 0.028)	-0.003 (-0.025, 0.018)	*0.68*
23:00-23:59	ref	0.002 (-0.019, 0.022)	0.006 (-0.013, 0.025)	0.019 (-0.001, 0.039)	0.006 (-0.016, 0.028)	*0.18*

[a] Expressed as beta coefficient and 95% CI derived from multiple linear regression models adjusted for age, sex, race/ethnicity, education, household income, marital status, smoking status, alcohol intake, total energy intake, sleep duration, sleep midpoint and total physical activity.
Abbreviations: β, beta coefficient; CI, confidence interval; NHANES, National Health and Nutrition Examination Survey; Q, quintile.

ESM Table 5 Associations [a] between average hourly physical activity and fasting insulin among NHANES (2011-2014) participants.

Hourly windows relative to sleep midpoint	Log-transformed fasting insulin (β (95% CI)), by quintiles of hourly physical activity					
	Q1	Q2	Q3	Q4	Q5	p_{trend}
0:00-0:59	ref	0.056 (-0.027, 0.139)	0.096 (0.004, 0.187)	0.068 (-0.028, 0.164)	0.126 (0, 0.253)	*0.03*
1:00-1:59	ref	0.004 (-0.085, 0.093)	0.074 (-0.033, 0.181)	0.100 (-0.012, 0.212)	-0.007 (-0.114, 0.101)	*0.35*
2:00-2:59	ref	-0.009 (-0.073, 0.054)	0.069 (-0.037, 0.174)	0.126 (0.037, 0.216)	0.039 (-0.093, 0.171)	*0.11*
3:00-3:59	ref	0.073 (-0.011, 0.158)	0.184 (0.068, 0.301)	0.107 (-0.018, 0.232)	0.180 (0.087, 0.274)	*0.002*
4:00-4:59	ref	0.027 (-0.065, 0.119)	0.104 (0.023, 0.184)	0.103 (0.015, 0.192)	0.159 (0.051, 0.267)	*0.002*
5:00-5:59	ref	0.002 (-0.106, 0.111)	0.084 (-0.032, 0.200)	0.036 (-0.055, 0.127)	0.097 (-0.017, 0.211)	*0.10*
6:00-6:59	ref	0 (-0.120, 0.121)	0.002 (-0.117, 0.121)	-0.029 (-0.163, 0.104)	-0.097 (-0.242, 0.047)	*0.14*
7:00-7:59	ref	-0.020 (-0.147, 0.108)	-0.061 (-0.199, 0.076)	-0.125 (-0.286, 0.036)	-0.182 (-0.353, -0.011)	*0.02*
8:00-8:59	ref	0.036 (-0.09, 0.163)	-0.03 (-0.154, 0.093)	-0.028 (-0.132, 0.075)	-0.078 (-0.255, 0.098)	*0.26*
9:00-9:59	ref	-0.052 (-0.179, 0.076)	-0.088 (-0.238, 0.062)	-0.112 (-0.248, 0.023)	-0.165 (-0.320, -0.011)	*0.02*
10:00-10:59	ref	0.024 (-0.072, 0.121)	0.048 (-0.071, 0.167)	-0.071 (-0.216, 0.074)	-0.08 (-0.239, 0.080)	*0.17*
11:00-11:59	ref	0.067 (-0.025, 0.158)	-0.024 (-0.123, 0.074)	-0.079 (-0.232, 0.074)	-0.067 (-0.223, 0.089)	*0.12*
12:00-12:59	ref	-0.014 (-0.122, 0.093)	-0.086 (-0.199, 0.027)	-0.068 (-0.211, 0.075)	-0.091 (-0.265, 0.084)	*0.25*
13:00-13:59	ref	-0.023 (-0.123, 0.076)	-0.055 (-0.162, 0.051)	-0.084 (-0.185, 0.017)	-0.068 (-0.183, 0.048)	*0.10*
14:00-14:59	ref	-0.021 (-0.143, 0.101)	-0.072 (-0.191, 0.047)	-0.070 (-0.226, 0.086)	-0.189 (-0.359, -0.019)	*0.04*
15:00-15:59	ref	-0.047 (-0.19, 0.097)	-0.083 (-0.204, 0.038)	-0.154 (-0.291, -0.017)	-0.181 (-0.339, -0.022)	*0.01*
16:00-16:59	ref	0.082 (-0.041, 0.204)	-0.04 (-0.146, 0.066)	-0.029 (-0.155, 0.098)	-0.018 (-0.156, 0.121)	*0.27*
17:00-17:59	ref	0.082 (-0.008, 0.172)	0.028 (-0.072, 0.128)	0.012 (-0.141, 0.165)	0.020 (-0.155, 0.196)	*0.79*
18:00-18:59	ref	0.043 (-0.038, 0.123)	0.004 (-0.098, 0.107)	0.024 (-0.093, 0.141)	0.026 (-0.117, 0.169)	*0.89*
19:00-19:59	ref	0.053 (-0.034, 0.140)	-0.052 (-0.159, 0.055)	-0.046 (-0.160, 0.067)	0.045 (-0.069, 0.158)	*0.89*
20:00-20:59	ref	0.050 (-0.068, 0.168)	0.081 (-0.053, 0.216)	0.054 (-0.079, 0.188)	0.095 (-0.070, 0.260)	*0.31*
21:00-21:59	ref	0.086 (0.005, 0.167)	0.123 (0.002, 0.243)	0.055 (-0.037, 0.147)	0.133 (0.021, 0.244)	*0.07*
22:00-22:59	ref	0.004 (-0.054, 0.062)	-0.067 (-0.151, 0.018)	0.056 (-0.029, 0.141)	0.104 (-0.041, 0.249)	*0.15*
23:00-23:59	ref	0.021 (-0.079, 0.121)	0.057 (-0.024, 0.139)	0.114 (0.002, 0.225)	0.167 (0.026, 0.309)	*0.01*

[a] Expressed as beta coefficient and 95% CI derived from multiple linear regression models adjusted for age, sex, race/ethnicity, education, household income, marital status, smoking status, alcohol intake, total energy intake, sleep duration, sleep midpoint and total physical activity.
Abbreviations: β, beta coefficient; CI, confidence interval; NHANES, National Health and Nutrition Examination Survey; Q, quintile.

ESM Table 6 Associations [a] between average hourly physical activity and HOMA-IR among NHANES (2011-2014) participants.

Hourly windows relative to sleep midpoint	Log-transformed fasting glucose (β (95% CI)), by quintiles of hourly physical activity					
	**Q1**	**Q2**	**Q3**	**Q4**	**Q5**	p_{trend}
0:00-0:59	ref	0.070 (-0.016, 0.156)	0.121 (0.024, 0.217)	0.090 (-0.016, 0.195)	0.133 (0.003, 0.263)	*0.02*
1:00-1:59	ref	-0.005 (-0.099, 0.09)	0.092 (-0.022, 0.206)	0.117 (-0.007, 0.242)	-0.003 (-0.113, 0.108)	*0.25*
2:00-2:59	ref	-0.010 (-0.085, 0.065)	0.068 (-0.045, 0.181)	0.140 (0.043, 0.237)	0.049 (-0.090, 0.188)	*0.09*
3:00-3:59	ref	0.099 (0.006, 0.192)	0.209 (0.080, 0.339)	0.122 (-0.015, 0.259)	0.207 (0.104, 0.311)	*0.002*
4:00-4:59	ref	0.029 (-0.070, 0.127)	0.123 (0.045, 0.200)	0.112 (0.009, 0.215)	0.177 (0.053, 0.302)	*0.003*
5:00-5:59	ref	0.011 (-0.107, 0.130)	0.100 (-0.024, 0.223)	0.040 (-0.058, 0.138)	0.098 (-0.025, 0.220)	*0.13*
6:00-6:59	ref	0.012 (-0.128, 0.153)	0.004 (-0.131, 0.140)	-0.025 (-0.174, 0.125)	-0.108 (-0.264, 0.048)	*0.13*
7:00-7:59	ref	-0.012 (-0.152, 0.127)	-0.058 (-0.214, 0.099)	-0.129 (-0.306, 0.048)	-0.192 (-0.380, -0.004)	*0.02*
8:00-8:59	ref	0.055 (-0.076, 0.187)	-0.023 (-0.151, 0.104)	-0.01 (-0.120, 0.099)	-0.057 (-0.248, 0.133)	*0.38*
9:00-9:59	ref	-0.034 (-0.182, 0.114)	-0.074 (-0.237, 0.089)	-0.098 (-0.244, 0.049)	-0.149 (-0.315, 0.017)	*0.03*
10:00-10:59	ref	0.047 (-0.059, 0.152)	0.073 (-0.057, 0.203)	-0.055 (-0.208, 0.098)	-0.057 (-0.224, 0.110)	*0.25*
11:00-11:59	ref	0.078 (-0.026, 0.182)	-0.026 (-0.133, 0.082)	-0.088 (-0.261, 0.085)	-0.071 (-0.237, 0.095)	*0.11*
12:00-12:59	ref	-0.015 (-0.139, 0.108)	-0.103 (-0.237, 0.032)	-0.076 (-0.237, 0.085)	-0.098 (-0.285, 0.089)	*0.25*
13:00-13:59	ref	-0.019 (-0.133, 0.095)	-0.062 (-0.186, 0.062)	-0.084 (-0.206, 0.038)	-0.060 (-0.189, 0.069)	*0.19*
14:00-14:59	ref	-0.025 (-0.166, 0.115)	-0.063 (-0.200, 0.074)	-0.077 (-0.254, 0.100)	-0.199 (-0.390, -0.007)	*0.05*
15:00-15:59	ref	-0.054 (-0.212, 0.103)	-0.093 (-0.232, 0.045)	-0.181 (-0.343, -0.020)	-0.202 (-0.377, -0.026)	*0.01*
16:00-16:59	ref	0.095 (-0.036, 0.227)	-0.049 (-0.168, 0.07)	-0.028 (-0.172, 0.115)	-0.025 (-0.178, 0.129)	*0.26*
17:00-17:59	ref	0.102 (0.008, 0.195)	0.056 (-0.056, 0.169)	0.038 (-0.120, 0.196)	0.036 (-0.150, 0.221)	*0.89*
18:00-18:59	ref	0.064 (-0.024, 0.153)	0.016 (-0.100, 0.131)	0.049 (-0.071, 0.169)	0.046 (-0.104, 0.195)	*0.73*
19:00-19:59	ref	0.061 (-0.032, 0.155)	-0.039 (-0.155, 0.077)	-0.038 (-0.16, 0.084)	0.067 (-0.057, 0.191)	*0.85*
20:00-20:59	ref	0.056 (-0.078, 0.190)	0.097 (-0.056, 0.250)	0.059 (-0.093, 0.210)	0.100 (-0.078, 0.279)	*0.33*
21:00-21:59	ref	0.097 (0.009, 0.185)	0.123 (-0.004, 0.250)	0.061 (-0.040, 0.163)	0.137 (0.021, 0.253)	*0.08*
22:00-22:59	ref	-0.005 (-0.068, 0.057)	-0.084 (-0.170, 0.003)	0.065 (-0.031, 0.161)	0.098 (-0.058, 0.253)	*0.18*
23:00-23:59	ref	0.023 (-0.091, 0.137)	0.065 (-0.022, 0.151)	0.134 (0.007, 0.260)	0.170 (0.016, 0.324)	*0.01*

[a] Expressed as beta coefficient and 95% CI derived from multiple linear regression models adjusted for age, sex, race/ethnicity, education, household income, marital status, smoking status, alcohol intake, total energy intake, sleep duration, sleep midpoint and total physical activity.
Abbreviations: β, beta coefficient; CI, confidence interval; HOMA-IR, homeostatic model assessment for insulin resistance; NHANES, National Health and Nutrition Examination Survey; Q, quintile.

ESM Table 7 Associations [a] between average hourly physical activity and prevalent diabetes [b] among NHANES (2011-2014) participants, by sex.

Hourly windows relative to sleep midpoint	Diabetes, OR_{Q5vQ1} (95% CI), per each quintile increase of hourly physical activity	
	**Women**	**Men**
0:00-0:59	1.13 (1.03, 1.26)	1.23 (1.13, 1.33)
1:00-1:59	1.14 (1.05, 1.24)	1.14 (1.03, 1.25)
2:00-2:59	1.15 (1.06, 1.25)	1.15 (1.06, 1.24)
3:00-3:59	1.23 (1.13, 1.35)	1.18 (1.05, 1.32)
4:00-4:59	1.13 (1.03, 1.25)	1.13 (1.02, 1.24)
5:00-5:59	1.05 (0.95, 1.17)	0.96 (0.86, 1.07)
6:00-6:59	1.01 (0.90, 1.12)	0.87 (0.78, 0.98)
7:00-7:59	0.93 (0.81, 1.07)	0.90 (0.77, 1.05)
8:00-8:59	0.81 (0.71, 0.93)	0.95 (0.83, 1.10)
9:00-9:59	0.94 (0.80, 1.10)	0.90 (0.78, 1.02)
10:00-10:59	1.05 (0.89, 1.23)	0.93 (0.83, 1.04)
11:00-11:59	0.85 (0.73, 0.97)	0.90 (0.79, 1.02)
12:00-12:59	0.83 (0.70, 0.98)	0.86 (0.77, 0.96)
13:00-13:59	0.83 (0.74, 0.93)	0.83 (0.73, 0.96)
14:00-14:59	0.82 (0.71, 0.94)	0.83 (0.73, 0.96)
15:00-15:59	0.87 (0.77, 1.00)	0.84 (0.70, 1.01)
16:00-16:59	0.95 (0.83, 1.08)	0.87 (0.77, 0.98)
17:00-17:59	1.06 (0.91, 1.24)	1.02 (0.89, 1.16)
18:00-18:59	1.05 (0.92, 1.21)	1.00 (0.89, 1.13)
19:00-19:59	1.00 (0.90, 1.13)	1.05 (0.88, 1.25)
20:00-20:59	1.03 (0.92, 1.16)	1.06 (0.95, 1.20)
21:00-21:59	1.02 (0.94, 1.11)	1.11 (0.99, 1.24)
22:00-22:59	1.01 (0.94, 1.09)	1.22 (1.12, 1.32)
23:00-23:59	1.12 (1.02, 1.23)	1.27 (1.18, 1.37)

[a] Expressed as OR and 95% CI per each quintile increase in hourly physical activity, derived from multiple logistic regression models adjusted for age, sex, race/ethnicity, education, household income, marital status, smoking status, alcohol intake, total energy intake, sleep duration, sleep midpoint and total physical activity.

[b] Defined as $HbA_{1c} \geq 6.5\%$ or self-reported diagnosis of diabetes.

Abbreviations: CI, confidence interval; HbA_{1c}, haemoglobin A1c; NHANES, National Health and Nutrition Examination Survey; OR, odds ratio.

ESM Table 8 Associations [a] between average hourly physical activity and prevalent diabetes [b] among NHANES (2011-2014) participants, by age.

Hourly windows relative to sleep midpoint	Diabetes, OR_{Q5vQ1} (95% CI), per each quintile increase of hourly physical activity	
	Age < 65	Age ≥ 65
0:00-0:59	1.17 (1.09, 1.25)	1.18 (1.06, 1.31)
1:00-1:59	1.15 (1.06, 1.25)	1.09 (1.00, 1.19)
2:00-2:59	1.17 (1.07, 1.27)	1.13 (1.02, 1.25)
3:00-3:59	1.29 (1.17, 1.42)	1.06 (0.98, 1.15)
4:00-4:59	1.18 (1.08, 1.31)	0.99 (0.89, 1.10)
5:00-5:59	1.00 (0.90, 1.11)	1.00 (0.88, 1.13)
6:00-6:59	0.93 (0.84, 1.03)	0.99 (0.88, 1.11)
7:00-7:59	0.88 (0.79, 0.99)	0.99 (0.84, 1.17)
8:00-8:59	0.85 (0.78, 0.93)	0.90 (0.77, 1.06)
9:00-9:59	0.92 (0.82, 1.02)	0.84 (0.68, 1.04)
10:00-10:59	0.98 (0.86, 1.12)	0.96 (0.82, 1.14)
11:00-11:59	0.83 (0.73, 0.94)	0.86 (0.73, 1.01)
12:00-12:59	0.84 (0.71, 0.99)	0.74 (0.65, 0.84)
13:00-13:59	0.80 (0.73, 0.87)	0.84 (0.67, 1.04)
14:00-14:59	0.79 (0.70, 0.90)	0.87 (0.73, 1.04)
15:00-15:59	0.86 (0.76, 0.97)	0.83 (0.68, 1.01)
16:00-16:59	0.91 (0.81, 1.02)	0.86 (0.73, 1.02)
17:00-17:59	1.05 (0.92, 1.21)	1.01 (0.86, 1.19)
18:00-18:59	1.08 (0.98, 1.20)	1.01 (0.87, 1.16)
19:00-19:59	1.10 (0.99, 1.23)	0.98 (0.87, 1.09)
20:00-20:59	1.05 (0.94, 1.17)	1.09 (0.95, 1.26)
21:00-21:59	1.05 (0.95, 1.17)	1.06 (0.98, 1.15)
22:00-22:59	1.12 (1.03, 1.21)	1.08 (0.98, 1.19)
23:00-23:59	1.19 (1.10, 1.29)	1.19 (1.05, 1.34)

[a] Expressed as OR and 95% CI per each quintile increase in hourly physical activity, derived from multiple logistic regression models adjusted for age, sex, race/ethnicity, education, household income, marital status, smoking status, alcohol intake, total energy intake, sleep duration, sleep midpoint and total physical activity.

[b] Defined as HbA_{1c}≥6.5% or self-reported diagnosis of diabetes.

Abbreviations: CI, confidence interval; HbA_{1c}, haemoglobin A1c; NHANES, National Health and Nutrition Examination Survey; OR, odds ratio.

ESM Table 9 Associations[a] between average hourly physical activity and prevalent diabetes[b] among NHANES (2011-2014) participants, by race/ethnicity.

Hourly windows relative to sleep midpoint	Diabetes, OR_{Q5vQ1} (95% CI), per each quintile increase of hourly physical activity		
	**NH White**	**NH Black**	**Hispanic[c]**
0:00-0:59	1.17 (1.09, 1.26)	1.09 (0.97, 1.22)	1.17 (1.04, 1.32)
1:00-1:59	1.07 (0.98, 1.17)	1.18 (1.07, 1.30)	1.23 (1.12, 1.36)
2:00-2:59	1.12 (1.05, 1.20)	1.15 (1.05, 1.26)	1.15 (1.02, 1.31)
3:00-3:59	1.22 (1.10, 1.35)	1.11 (0.97, 1.27)	1.09 (0.96, 1.24)
4:00-4:59	1.12 (1.01, 1.26)	1.09 (0.95, 1.25)	1.13 (1.00, 1.26)
5:00-5:59	1.01 (0.91, 1.11)	0.95 (0.84, 1.08)	1.11 (0.96, 1.28)
6:00-6:59	1.00 (0.90, 1.11)	0.91 (0.79, 1.04)	0.91 (0.78, 1.05)
7:00-7:59	0.95 (0.82, 1.12)	0.85 (0.74, 0.97)	0.93 (0.80, 1.06)
8:00-8:59	0.90 (0.78, 1.04)	0.79 (0.69, 0.91)	0.87 (0.74, 1.01)
9:00-9:59	0.95 (0.81, 1.11)	0.91 (0.81, 1.03)	0.85 (0.71, 1.02)
10:00-10:59	1.05 (0.90, 1.23)	0.95 (0.83, 1.10)	0.81 (0.70, 0.93)
11:00-11:59	0.87 (0.76, 1.01)	0.89 (0.77, 1.03)	0.83 (0.71, 0.98)
12:00-12:59	0.79 (0.67, 0.95)	0.82 (0.70, 0.97)	1.01 (0.84, 1.21)
13:00-13:59	0.83 (0.73, 0.94)	0.87 (0.77, 0.98)	0.93 (0.82, 1.05)
14:00-14:59	0.78 (0.65, 0.94)	0.84 (0.71, 1.00)	0.96 (0.81, 1.14)
15:00-15:59	0.83 (0.67, 1.00)	0.79 (0.62, 1.01)	1.05 (0.89, 1.23)
16:00-16:59	0.90 (0.76, 1.06)	0.87 (0.72, 1.05)	1.04 (0.89, 1.21)
17:00-17:59	1.09 (0.89, 1.34)	0.80 (0.67, 0.95)	1.12 (0.97, 1.30)
18:00-18:59	1.03 (0.90, 1.17)	0.93 (0.79, 1.09)	1.16 (1.04, 1.30)
19:00-19:59	1.00 (0.88, 1.14)	0.97 (0.85, 1.12)	1.05 (0.91, 1.22)
20:00-20:59	1.02 (0.91, 1.16)	1.04 (0.90, 1.21)	1.05 (0.89, 1.24)
21:00-21:59	1.07 (0.94, 1.22)	1.03 (0.90, 1.18)	0.96 (0.83, 1.11)
22:00-22:59	1.08 (0.99, 1.18)	1.10 (0.99, 1.22)	1.01 (0.93, 1.11)
23:00-23:59	1.17 (1.07, 1.28)	1.18 (1.06, 1.32)	1.13 (0.97, 1.32)

[a] Expressed as OR and 95% CI per each quintile increase in hourly physical activity, derived from multiple logistic regression models adjusted for age, sex, race/ethnicity, education, household income, marital status, smoking status, alcohol intake, total energy intake, sleep duration, sleep midpoint and total physical activity.

[b] Defined as HbA_{1c}≥6.5% or self-reported diagnosis of diabetes.

[c] Including both Mexican and other Hispanic groups.

Abbreviations: CI, confidence interval; HbA_{1c}, haemoglobin A1c; NH, non-Hispanic; NHANES, National Health and Nutrition Examination Survey; OR, odds ratio.

ESM Table 10 Associations [a] between average hourly physical activity and prevalent diabetes [b] among NHANES (2011-2014) participants, by chronotype [c].

Hourly windows relative to sleep midpoint	Diabetes, OR_{Q5vQ1} (95% CI), per each quintile increase of hourly physical activity	
	**Midpoint < 03:15**	**Midpoint ≥ 03:15**
0:00-0:59	1.15 (1.03, 1.26)	1.25 (1.13, 1.38)
1:00-1:59	1.09 (0.99, 1.19)	1.18 (1.06, 1.32)
2:00-2:59	1.11 (1.02, 1.20)	1.21 (1.11, 1.31)
3:00-3:59	1.25 (1.14, 1.37)	1.16 (1.04, 1.29)
4:00-4:59	1.11 (0.99, 1.24)	1.14 (1.01, 1.29)
5:00-5:59	0.99 (0.86, 1.14)	0.99 (0.87, 1.13)
6:00-6:59	0.94 (0.82, 1.08)	0.91 (0.81, 1.03)
7:00-7:59	0.96 (0.84, 1.10)	0.89 (0.78, 1.02)
8:00-8:59	0.97 (0.85, 1.11)	0.79 (0.65, 0.96)
9:00-9:59	0.95 (0.84, 1.07)	0.85 (0.73, 0.98)
10:00-10:59	1.03 (0.88, 1.20)	0.93 (0.83, 1.04)
11:00-11:59	0.85 (0.76, 0.96)	0.93 (0.79, 1.10)
12:00-12:59	0.92 (0.78, 1.09)	0.75 (0.66, 0.86)
13:00-13:59	0.93 (0.84, 1.03)	0.75 (0.64, 0.88)
14:00-14:59	0.85 (0.72, 0.99)	0.82 (0.70, 0.96)
15:00-15:59	0.86 (0.72, 1.02)	0.85 (0.75, 0.96)
16:00-16:59	0.90 (0.80, 1.01)	0.90 (0.79, 1.03)
17:00-17:59	1.04 (0.87, 1.25)	1.00 (0.88, 1.14)
18:00-18:59	1.09 (0.94, 1.25)	0.91 (0.81, 1.02)
19:00-19:59	1.08 (0.98, 1.19)	0.94 (0.82, 1.08)
20:00-20:59	1.01 (0.87, 1.18)	1.07 (0.95, 1.21)
21:00-21:59	0.98 (0.89, 1.09)	1.18 (1.00, 1.38)
22:00-22:59	1.03 (0.93, 1.13)	1.23 (1.10, 1.39)
23:00-23:59	1.10 (1.01, 1.20)	1.33 (1.18, 1.50)

[a] Expressed as OR and 95% CI per each quintile increase in hourly physical activity, derived from multiple logistic regression models adjusted for age, sex, race/ethnicity, education, household income, marital status, smoking status, alcohol intake, total energy intake, sleep duration, sleep midpoint and total physical activity.
[b] Defined as HbA_{1c}≥6.5% or self-reported diagnosis of diabetes.
[c] Defined by average sleep midpoint on weekend nights (Friday and Saturday night).
Abbreviations: CI, confidence interval; HbA_{1c}, haemoglobin A1c; NHANES, National Health and Nutrition Examination Survey; OR, odds ratio.

ESM Table 11 Associations [a] between average hourly physical activity and prevalent diabetes [b] among NHANES (2011-2014) participants, by sleep duration.

Hourly windows relative to sleep midpoint	Diabetes, OR_{Q5vQ1} (95% CI), per each quintile increase of hourly physical activity	
	Sleep duration ≥ 7 hours	Sleep duration < 7hours
0:00-0:59	1.17 (1.08, 1.27)	1.21 (1.12, 1.30)
1:00-1:59	1.16 (1.07, 1.25)	1.14 (1.02, 1.27)
2:00-2:59	1.13 (1.05, 1.20)	1.21 (1.11, 1.32)
3:00-3:59	1.20 (1.08, 1.32)	1.24 (1.14, 1.36)
4:00-4:59	1.08 (0.97, 1.21)	1.24 (1.12, 1.38)
5:00-5:59	0.94 (0.87, 1.03)	1.08 (0.96, 1.23)
6:00-6:59	0.95 (0.85, 1.06)	0.93 (0.86, 1.01)
7:00-7:59	0.91 (0.81, 1.02)	0.90 (0.79, 1.03)
8:00-8:59	0.92 (0.79, 1.08)	0.83 (0.73, 0.96)
9:00-9:59	0.95 (0.78, 1.17)	0.89 (0.81, 0.98)
10:00-10:59	1.02 (0.84, 1.23)	0.96 (0.85, 1.09)
11:00-11:59	0.86 (0.72, 1.03)	0.87 (0.76, 0.99)
12:00-12:59	0.81 (0.69, 0.94)	0.86 (0.74, 0.98)
13:00-13:59	0.86 (0.73, 1.02)	0.80 (0.72, 0.90)
14:00-14:59	0.81 (0.68, 0.98)	0.83 (0.73, 0.94)
15:00-15:59	0.86 (0.72, 1.03)	0.85 (0.75, 0.96)
16:00-16:59	1.04 (0.93, 1.17)	0.80 (0.70, 0.91)
17:00-17:59	1.15 (0.97, 1.36)	0.95 (0.83, 1.09)
18:00-18:59	0.99 (0.87, 1.12)	1.06 (0.91, 1.22)
19:00-19:59	0.89 (0.78, 1.02)	1.16 (1.01, 1.34)
20:00-20:59	0.89 (0.79, 1.00)	1.24 (1.10, 1.40)
21:00-21:59	0.97 (0.86, 1.09)	1.19 (1.08, 1.32)
22:00-22:59	1.01 (0.91, 1.12)	1.21 (1.13, 1.30)
23:00-23:59	1.15 (1.06, 1.26)	1.25 (1.12, 1.39)

[a] Expressed as OR and 95% CI per each quintile increase in hourly physical activity, derived from multiple logistic regression models adjusted for age, sex, race/ethnicity, education, household income, marital status, smoking status, alcohol intake, total energy intake, sleep duration, sleep midpoint and total physical activity.

[b] Defined as HbA_{1c}≥6.5% or self-reported diagnosis of diabetes.

[c] Defined by average sleep midpoint on weekend nights (Friday and Saturday night).

Abbreviations: CI, confidence interval; HbA_{1c}, haemoglobin A1c; NHANES, National Health and Nutrition Examination Survey; OR, odds ratio.

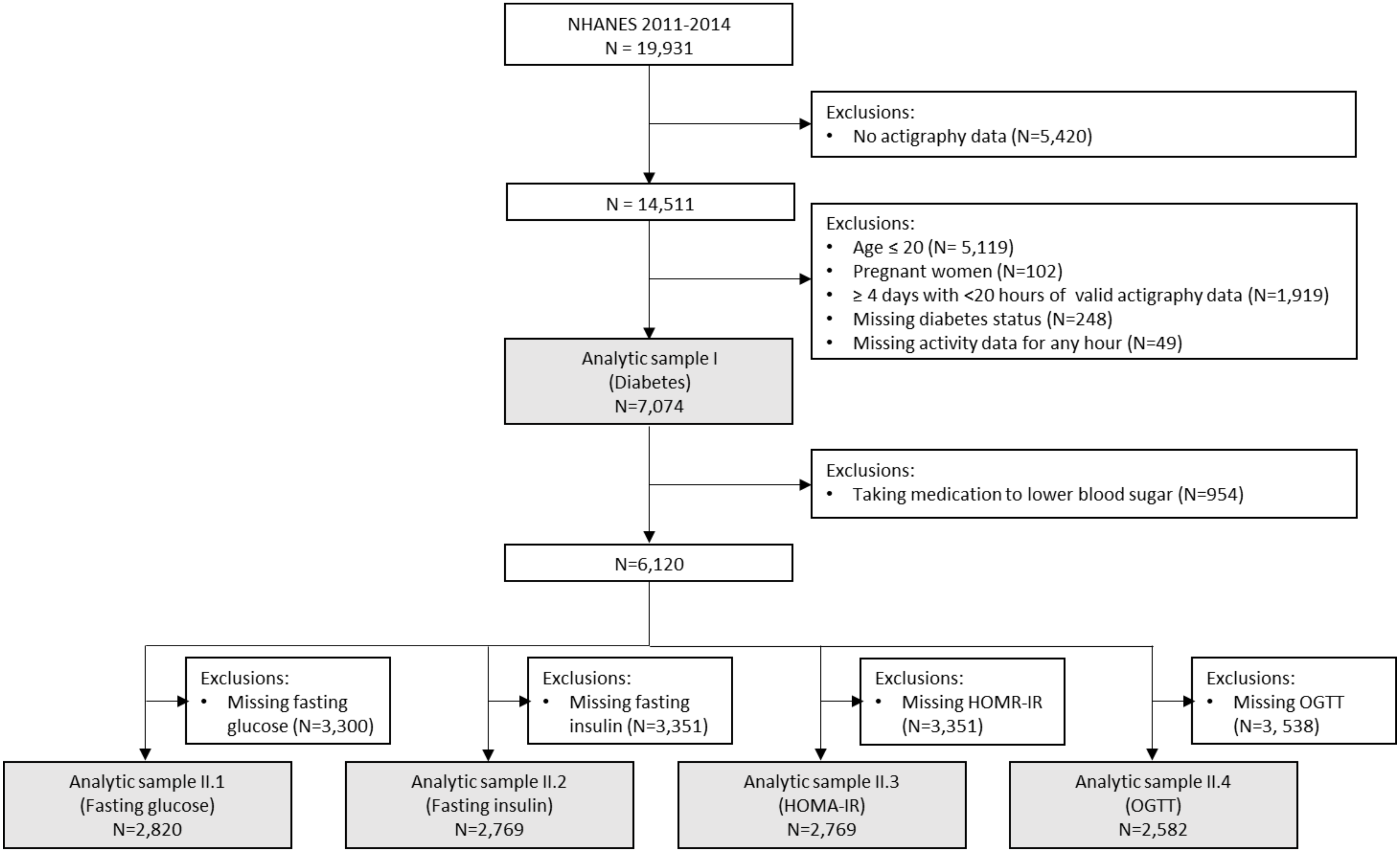

ESM Figure 1. Flow chart of study sample seleciton. Abbreviations: HOMA-IR, homeostatic model assessment for insulin resistance; OGTT, 2-hour oral glucose tolerance test

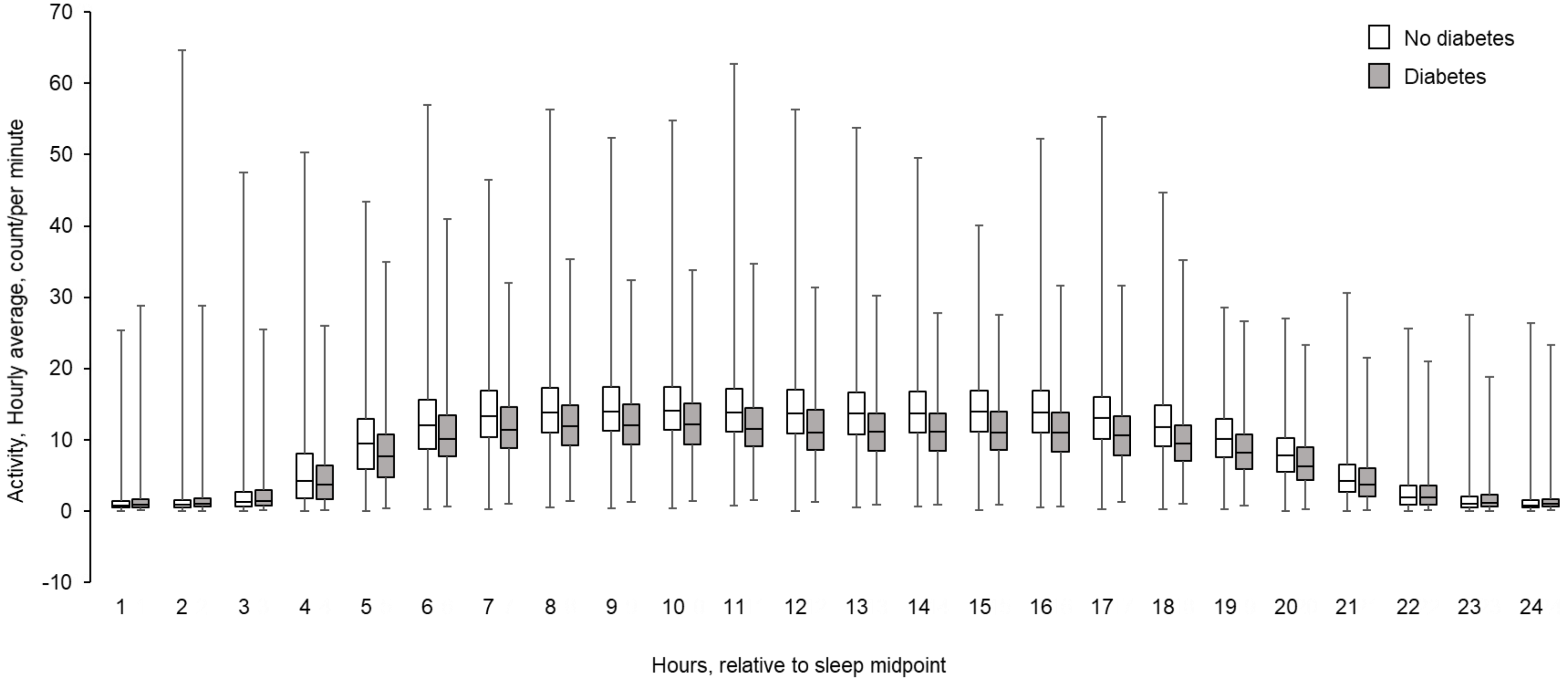

ESM Figure 2. Average hourly physical activity (count/minute) patterns among NHANES (2011-2014) participants with and without diabetes. Each hourly window was determined as relative to the average sleep midpoint of the individual (e.g., hour 1 is within the first hour after the sleep midpoint). The activity level was measured by the Monitor-Independent Movement Summary (MIMS) units. The box plot shows the population median and IQR. Abbreviations: IQR, interquartile range; NHANES, National Health and Nutrition Examination Survey.

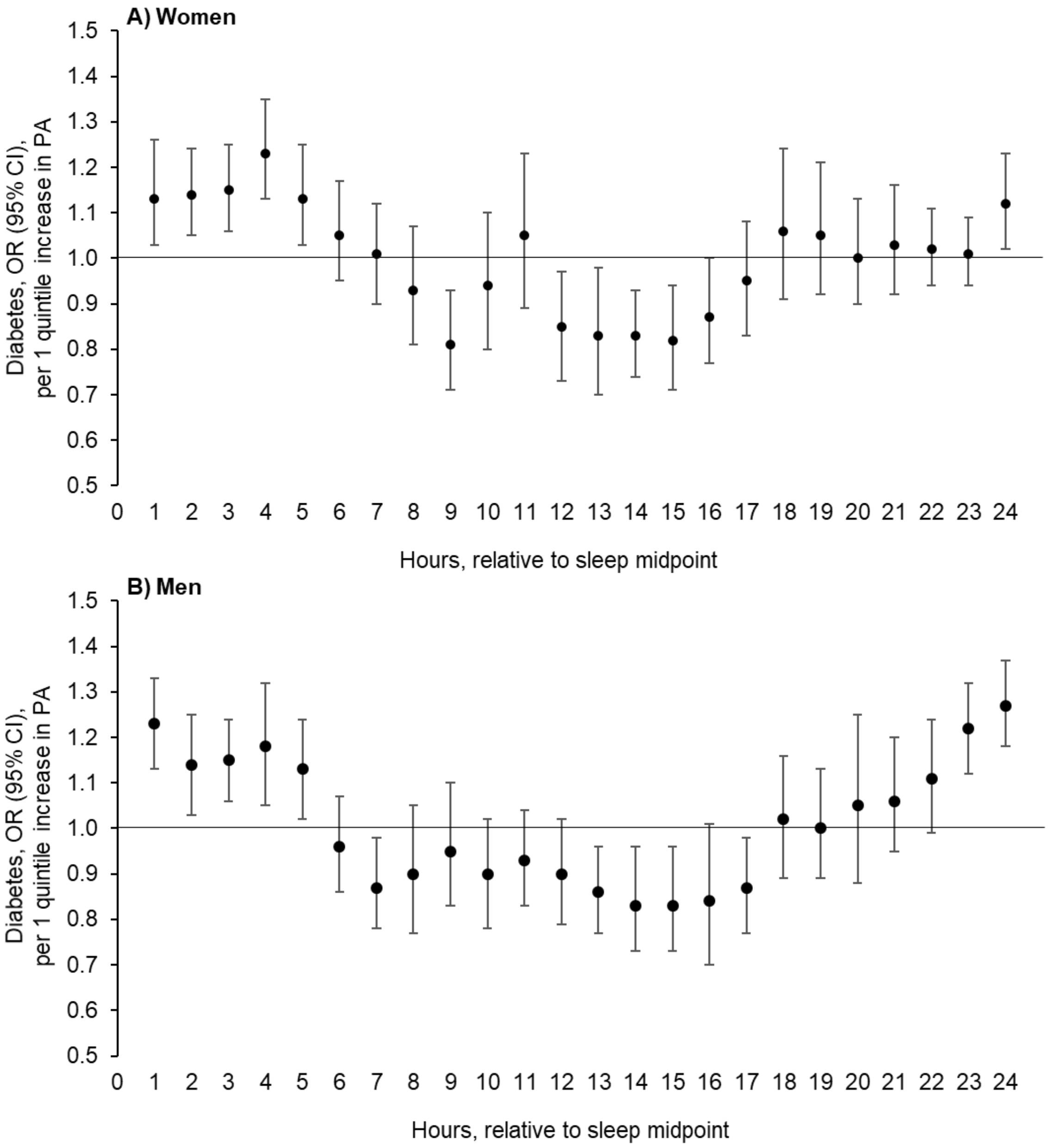

ESM Figure 3. Associations [a] between average hourly physical activity and prevalent diabetes [b] among NHANES (2011-2014) participants, by sex. Each hourly window was determined as relative to the average sleep midpoint of the individual (e.g., hour 1 is within the first hour after the sleep midpoint). [a] Expressed as OR and 95% CI per each quintile increase in hourly physical activity, derived from multiple logistic regression models adjusted for age (continuous) and sex (men, women), race/ethnicity (non-Hispanic white, NH black, Hispanic, others), education (less than high school, high school graduate, some college, college graduate or above), household income (<$20k, $20k-44.9k, $45k-74.9k, $75k+), marital status (married, not married), smoking (current smoker, former smoker, never smoker or less than 100 cigarettes in life), alcohol consumption (<1 drink/week, 1 drink/week-<1 drink/day, 1+ drink/day), body mass index (<18.5, 18.5-<25, 25-<30, 30+), total energy intake (continuous), sleep duration (<7, 7-9, >9 hours), sleep midpoint (continuous), and daily total physical activity (continuous). [b] Defined as $HbA_{1c} \geqslant$

48 mmol/mol (6.5%) or self-reported diagnosis of diabetes. Abbreviations: CI, confidence interval; HbA_{1c}, haemoglobin A1c; NHANES, National Health and Nutrition Examination Survey; OR, odds ratio; Q, quintile.

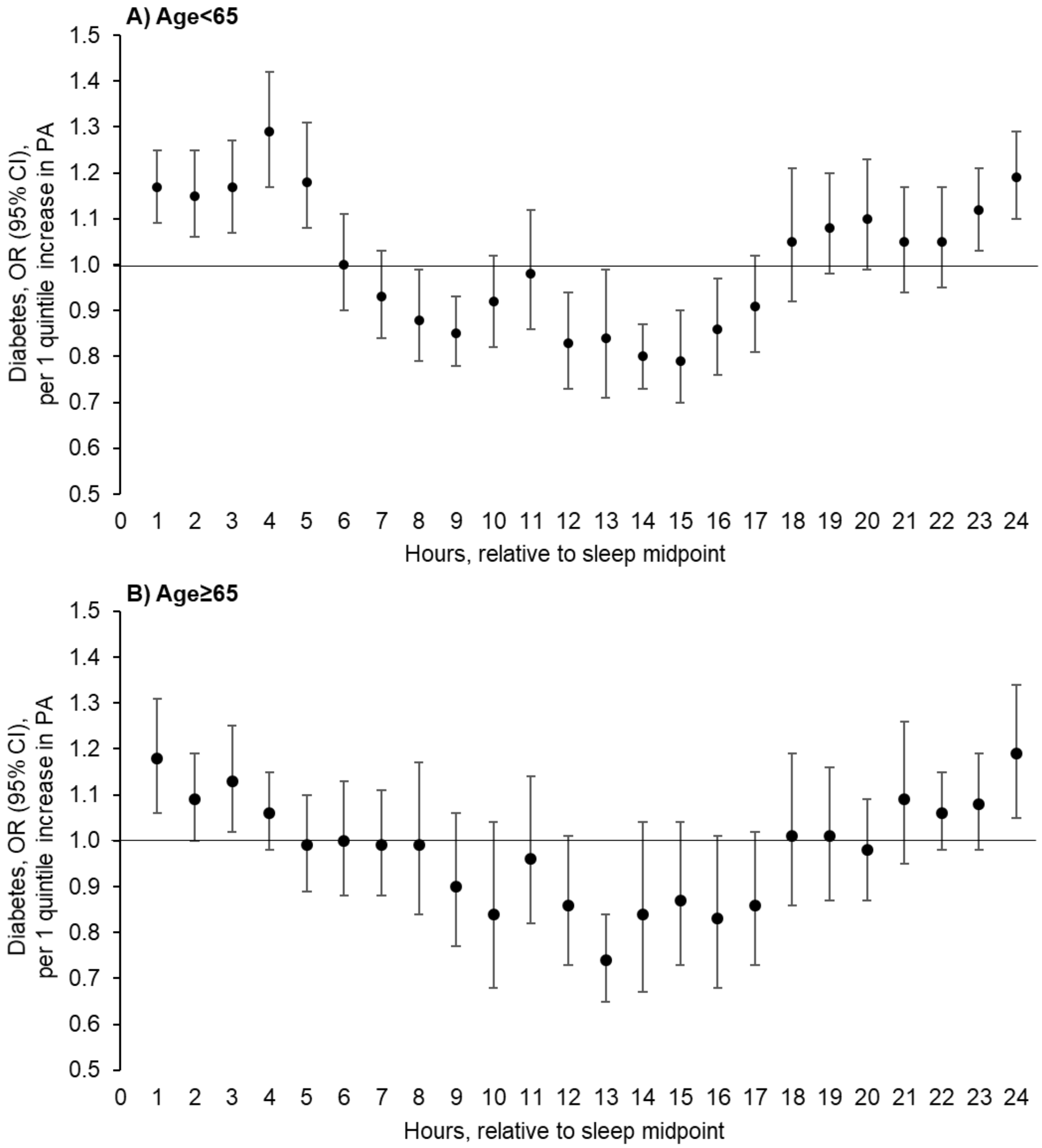

ESM Figure 4. Associations [a] between average hourly physical activity and diabetes [b] among NHANES (2011-2014) participants, by age. Each hourly window was determined as relative to the average sleep midpoint of the individual (e.g., hour 1 is within the first hour after the sleep midpoint). [a] Expressed as OR and 95% CI per each quintile increase in hourly physical activity, derived from multiple logistic regression models adjusted for age (continuous) and sex (men, women), race/ethnicity (non-Hispanic white, NH black, Hispanic, others), education (less than high school, high school graduate, some college, college graduate or above), household income (<$20k, $20k-44.9k, $45k-74.9k, $75k+), marital status (married, not married), smoking (current smoker, former smoker, never smoker or less than 100 cigarettes in life), alcohol consumption (<1 drink/week, 1 drink/week-<1 drink/day, 1+ drink/day), body mass index (<18.5, 18.5-<25, 25-<30, 30+), total energy intake (continuous), sleep duration (<7, 7-9, >9 hours), sleep midpoint (continuous), and daily total physical activity (continuous). [b] Defined as HbA1c≥

48 mmol/mol (6.5%)or self-reported diagnosis of diabetes. Abbreviations: CI, confidence interval; HbA1c, haemoglobin A1c; NHANES, National Health and Nutrition Examination Survey; OR, odds ratio; Q, quintile.

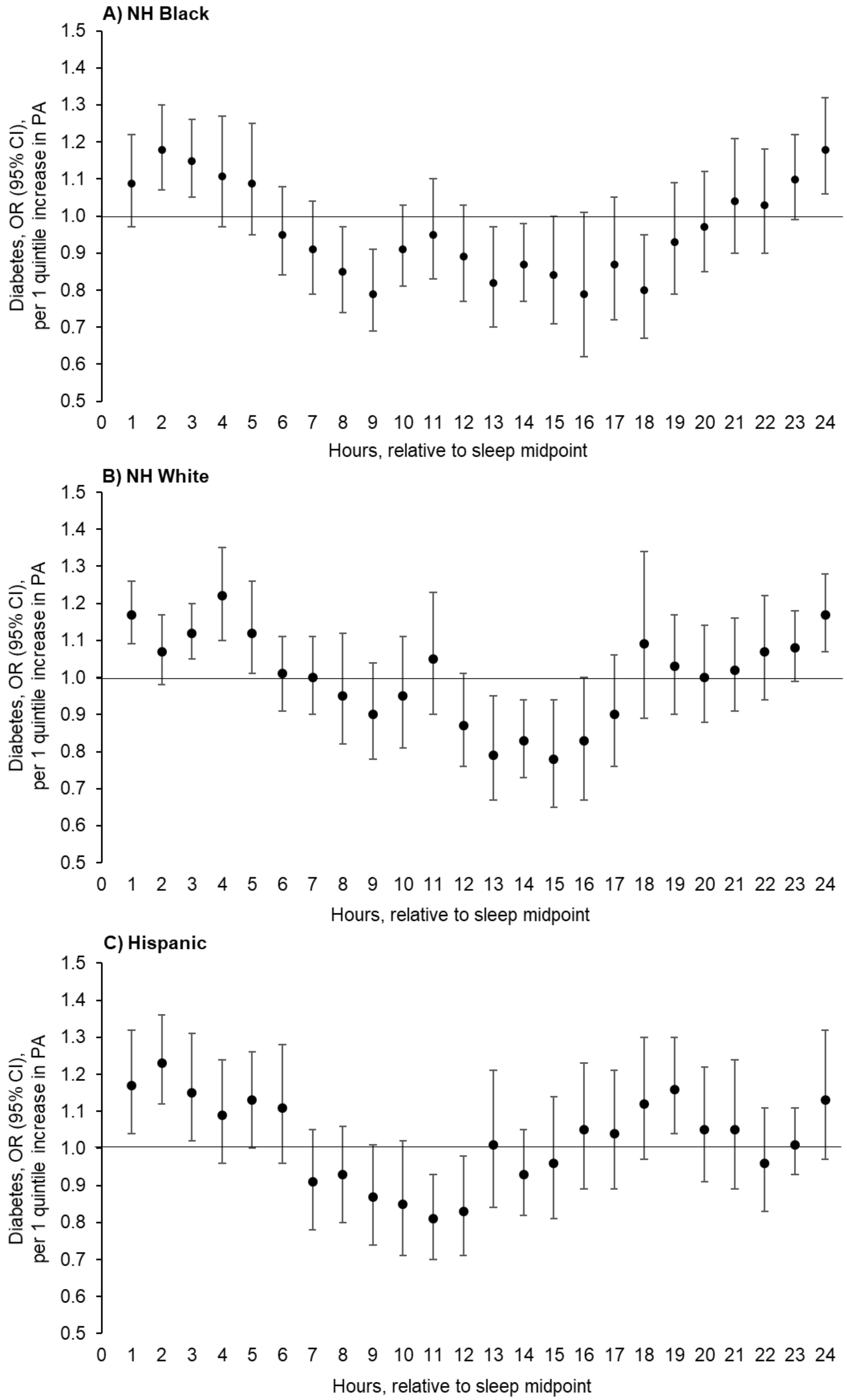
A) NH Black
B) NH White
C) Hispanic
Diabetes, OR (95% CI),
per 1 quintile increase in PA
Hours, relative to sleep midpoint
1.5
1.4
1.3
1.2
1.1
1.0
0.9
0.8
0.7
0.6
0.5
0 1 2 3 4 5 6 7 8 9 10 11 12 13 14 15 16 17 18 19 20 21 22 23 24

ESM Figure 5. Associations [a] between average hourly physical activity and diabetes [b] among NHANES (2011-2014) participants, by race/ethnicity. Each hourly window was determined as relative to the average sleep midpoint of the individual (e.g., hour 1 is within the first hour after the sleep midpoint). [a] Expressed as OR and 95% CI per each quintile increase in hourly physical activity, derived from multiple logistic regression models adjusted for age (continuous) and sex (men, women), race/ethnicity (non-Hispanic white, NH black, Hispanic, others), education (less than high school, high school graduate, some college, college graduate or above), household income (<$20k, $20k-44.9k, $45k-74.9k, $75k+), marital status (married, not married), smoking (current smoker, former smoker, never smoker or less than 100 cigarettes in life), alcohol consumption (<1 drink/week, 1 drink/week-<1 drink/day, 1+ drink/day), body mass index (<18.5, 18.5-<25, 25-<30, 30+), total energy intake (continuous), sleep duration (<7, 7-9, >9 hours), sleep midpoint (continuous), and daily total physical activity (continuous). [b] Defined as HbA1c$\geqslant$ 48 mmol/mol (6.5%)or self-reported diagnosis of diabetes. Abbreviations: CI, confidence interval; HbA1c, haemoglobin A1c; NH, non-Hispanic; NHANES, National Health and Nutrition Examination Survey; OR, odds ratio; Q, quintile.

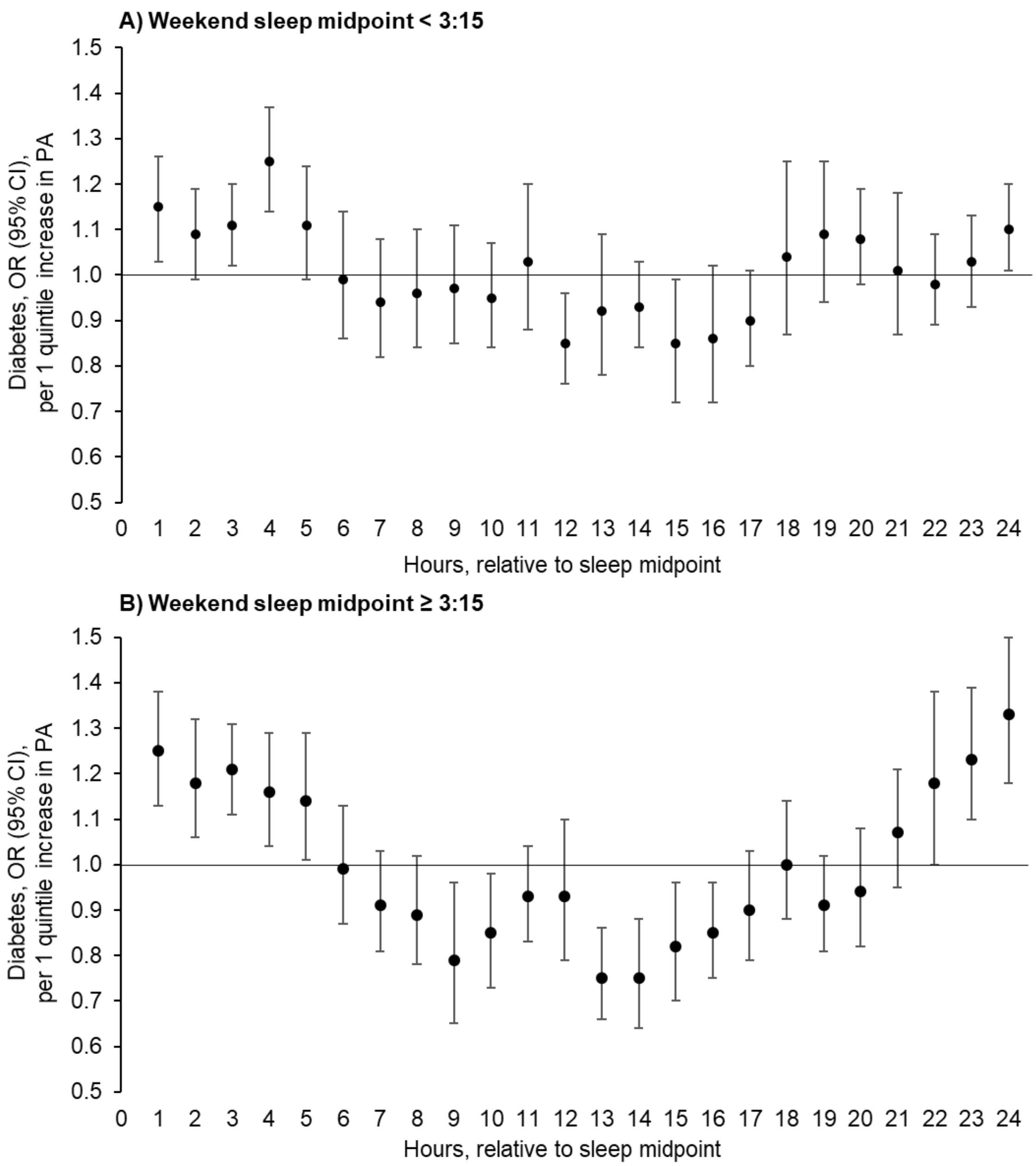

ESM Figure 6. Associations [a] between average hourly physical activity and diabetes [b] among NHANES (2011-2014) participants, by chronotype [c]. Each hourly window was determined as relative to the average sleep midpoint of the individual (e.g., hour 1 is within the first hour after the sleep midpoint). [a] Expressed as OR and 95% CI per each quintile increase in hourly physical activity, derived from multiple logistic regression models adjusted for age (continuous) and sex (men, women), race/ethnicity (non-Hispanic white, NH black, Hispanic, others), education (less than high school, high school graduate, some college, college graduate or above), household income (<$20k, $20k-44.9k, $45k-74.9k, $75k+), marital status (married, not married), smoking (current smoker, former smoker, never smoker or less than 100 cigarettes in life), alcohol consumption (<1 drink/week, 1 drink/week-<1 drink/day, 1+ drink/day), body mass index (<18.5, 18.5-<25, 25-<30, 30+), total energy intake (continuous), sleep duration (<7, 7-9, >9 hours), sleep midpoint (continuous), and daily total physical activity (continuous). [b] Defined as HbA1c≥

48 mmol/mol (6.5%) or self-reported diagnosis of diabetes. [c] Defined based on weekend sleep midpoint. Abbreviations: CI, confidence interval; HbA1c, haemoglobin A1c; NHANES, National Health and Nutrition Examination Survey; OR, odds ratio; Q, quintile.

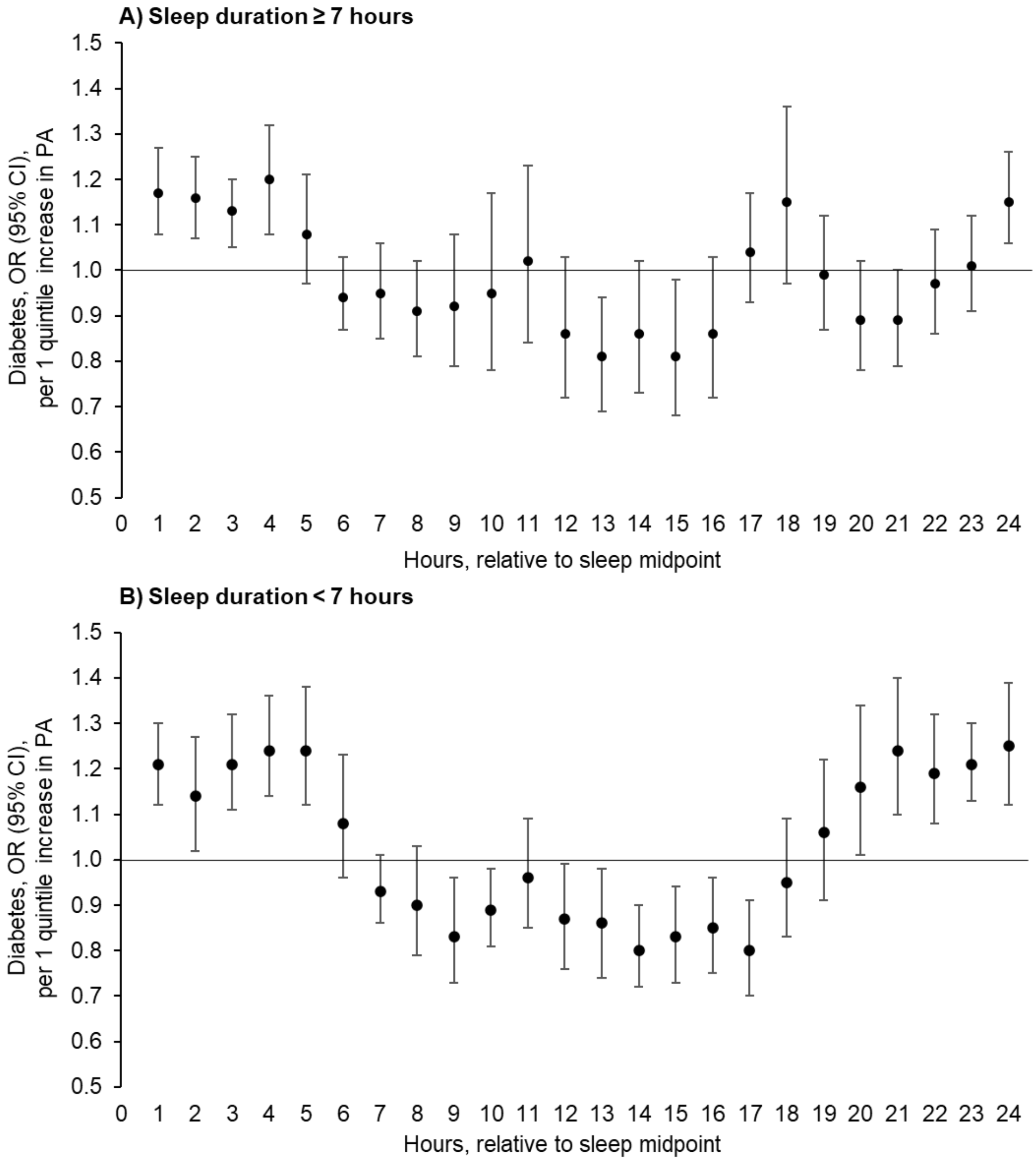

ESM Figure 7. Associations [a] between average hourly physical activity and diabetes [b] among NHANES (2011-2014) participants, by sleep duration. Each hourly window was determined as relative to the average sleep midpoint of the individual (e.g., hour 1 is within the first hour after the sleep midpoint). [a] Expressed as OR and 95% CI per each quintile increase in hourly physical activity, derived from multiple logistic regression models adjusted for age (continuous) and sex (men, women), race/ethnicity (non-Hispanic white, NH black, Hispanic, others), education (less than high school, high school graduate, some college, college graduate or above), household income (<$20k, $20k-44.9k, $45k-74.9k, $75k+), marital status (married, not married), smoking (current smoker, former smoker, never smoker or less than 100 cigarettes in life), alcohol consumption (<1 drink/week, 1 drink/week-<1 drink/day, 1+ drink/day), body mass index (<18.5, 18.5-<25, 25-<30, 30+), total energy intake (continuous), sleep duration (<7, 7-9, >9 hours), sleep midpoint (continuous), and daily total physical activity (continuous). [b] Defined as HbA1c≥

48 mmol/mol (6.5%) or self-reported diagnosis of diabetes. [c] Defined based on weekend sleep midpoint. Abbreviations: CI, confidence interval; HbA1c, haemoglobin A1c; NHANES, National Health and Nutrition Examination Survey; OR, odds ratio; Q, quintile.
